# Cheminformatics Bioprospection of Sunflower Seeds’ Oils against Quorum Sensing System of *Pseudomonas aeruginosa*

**DOI:** 10.3390/antibiotics12030504

**Published:** 2023-03-02

**Authors:** Nosipho Wendy S’thebe, Jamiu Olaseni Aribisala, Saheed Sabiu

**Affiliations:** Department of Biotechnology and Food Science, Faculty of Applied Science, Durban University of Technology, Durban 4001, South Africa

**Keywords:** autoinducer, biofilm, molecular dynamics simulation, *Pseudomonas aeruginosa*, quorum sensing

## Abstract

Clinically significant pathogens such as *Pseudomonas aeruginosa* evade the effects of antibiotics using quorum sensing (QS) systems, making antimicrobial resistance (AMR) a persistent and potentially fatal global health issue. Hence, QS has been identified as a novel therapeutic target for identifying novel drug candidates against *P. aeruginosa*, and plant-derived products, including essential oils, have been demonstrated as effective QS modulators. This study assessed the antipathogenic efficacy of essential oils from two sunflower cultivars (AGSUN 5102 CLP and AGSUN 5106 CLP) against *P. aeruginosa* ATCC 27853 in vitro and in silico. At the sub-inhibitory concentrations, both AGSUN 5102 CLP (62.61%) and AGSUN 5106 CLP (59.23%) competed favorably with cinnamaldehyde (60.74%) and azithromycin (65.15%) in suppressing the expression of QS-controlled virulence phenotypes and biofilm formation in *P. aeruginosa*. A further probe into the mechanism of anti-QS action of the oils over a 100-ns simulation period against Las QS system revealed that phylloquinone (−66.42 ± 4.63 kcal/mol), linoleic acid (−53.14 ± 3.53 kcal/mol), and oleic acid (−52.02 ± 3.91 kcal/mol) had the best affinity and structural compactness as potential modulators of LasR compared to cinnamaldehyde (−16.95 ± 1.75 kcal/mol) and azithromycin (−32.08 ± 10.54 kcal/mol). These results suggest that the identified compounds, especially phylloquinone, could be a possible LasR modulator and may represent a novel therapeutic alternative against infections caused by *P. aeruginosa*. As a result, phylloquinone could be further studied as a QS modulator and perhaps find utility in developing new therapeutics.

## 1. Introduction

Biofilm formation in *Pseudomonas aeruginosa* is believed to be responsible for 65% of *P. aeruginosa*-related deaths and poses a significant threat to individuals with cystic fibrosis (CF) due to antibiotic resistance [1]. Therefore, blocking biofilm formation in *P. aeruginosa* could help reduce mortality and curb antibiotic resistance. A biofilm consists of bacterial cells embedded in a matrix of extracellular polymeric substances (EPS) composed of lipids, macromolecules, DNA, exopolysaccharides, and proteins [2], rendering the organisms therein impermeable to antibiotics. Accordingly, the planktonic *P. aeruginosa* is 1000 times less resistant to antibiotics than biofilm-embedded *P. aeruginosa* [3,4,5]. Biofilm formation also allows *P. aeruginosa* to evade recognition by the host immune system [3], and this is coordinated by its quorum sensing (QS) system, which plays a crucial role in the formation, secretion, processing, and recognition of autoinducer (AL), a cell-to-cell communication molecule implicated in biofilm formation [6,7,8]. At specific density, as measured by the ambient AL concentration, the transcription of bacterial genes in the community becomes synchronized, allowing the organisms to act cooperatively. Thus, virulence factor secretion, swimming motility, secondary metabolite synthesis, biofilm development, and antibiotic resistance in the organism community are all controlled by ALs [9]. Consequently, disruption of *P. aeruginosa* QS-mediated signaling disrupts communication in the organism community, reducing pathogenicity and ultimately eradicating the organism from the host [10,11,12].

*Pseudomonas aeruginosa* has three main QS systems: LasI-LasR, RhlI-RhlR, and PQS-MvfR, all of which contribute to the formation of mature and differentiated biofilms [13,14,15]. However, the Las system regulates the expression of the other two systems with its autoinducer 3O-C12-HSL; thus, targeting the Las QS system could help prevent the expression of other QS systems [14,15,16]. In this regard, the molecular docking approach could help in the in silico screening of metabolites against the Las QS system due to its ability to identify lead metabolites from a library of compounds. Interestingly, the technique has been employed in the recent screening of metabolites against a bacterial QS system [17] and, thus, could serve as a less expensive approach to understanding compounds’ interactions with the QS system. However, due to the limitations of virtual screening relating to the lack of subsequent refinement (rescoring) and assessment of binding pose stability [18,19], further refinement and assessment of thermodynamic information are often encouraged using molecular mechanics/GB surface approach and molecular dynamics (MD) simulations [18,19], and in this study, these approaches were employed.

Several synthetic compounds such as azithromycin, erythromycin, levamisole, propranolol, and chloroquine have been shown to have anti-QS activities in the search for QS inhibitors via drug repurposing [20]. However, the side effects of these drugs and the increase in the frequency of *P. aeruginosa* strains with increased virulence following their use [11,21] have prompted researchers to investigate alternative QS inhibitors, such as plant-derived compounds. Many plants, such as *Pisum sativum* seedlings, *Citrus reticulata*, and *Syzygium aromaticum,* have been recently studied as potent QS modulators [11,14]. Interestingly, *Helianthus annuus* (sunflower) and its oil have been found to have nutritional and therapeutic relevance as anti-inflammatory, antimalarial, antiasthmatic, antioxidant, antitumor, and antibacterial agents [22]. However, despite the antibacterial significance of *H. annuus* and its derived products, there is a paucity of information on its antipathogenic activity through QS inhibition to date. Hence, this study evaluated the anti-QS activity of two sunflower seed oils using both in vitro and in silico techniques. In the in vitro assay, a biomonitor strain, *Chromobacterium violaceum,* was employed to understand and easily detect QS inhibition in *P. aeruginosa* due to *C. violaceum’s* ability to produce the purple pigment violacein [23]. Thus, in addition to identifying active Las modulators of *P. aeruginosa*, metabolites of sunflower seed oil were screened in silico against the main QS regulator in *C. violaceum,* the CviR QS system [23]. Identifying lead compounds from plant-derived products, such as oils, against important QS regulators of *P. aeruginosa* and *C. violaceum* could help to identify active anti-QS modulators that could fast-track novel drug discovery and development and contribute toward the increased commercial importance of plants.

## 2. Materials and Methods

### 2.1. Plant Collection, Preparation, and Oil Extraction

The two hybrids (AGSUN 5102 CLP and AGSUN 5106 CLP) of sunflower seeds used in this study were collected from the Agricultural Research Council, Potchefstroom, South Africa. The seeds were dehusked, washed, and oven-dried overnight at 30 °C to constant weight. Initially, the dried seeds were powdered using a mortar and pestle before 10 g of each was subjected to Soxhlet (Merck Sigma-Aldrich, Johannesburg, South Africa) extraction (50–55 °C) using 100 mL of n-hexane adopting a standard method [24]. After extraction, the remaining n-hexane was evaporated using a Heidolph Hei-VAP core rotary evaporator (ProfiLab24, Landsberger, Berlin) maintained at 40 °C, leaving only the extracted oil in the flask. The oil extracted was stored in an air-tight container and covered with aluminum foil to prevent auto-oxidation due to sunlight exposure. The oils were stored at 4 °C for further analysis. For the in vitro assays, stock solutions of oils were re-diluted to the required concentrations using 2% dimethyl sulfoxide (DMSO).

### 2.2. In Vitro Analyses

#### 2.2.1. Bacterial Strains and Growth Conditions

For this study, *P. aeruginosa* ATCC 27853 and *C. violaceum* ATCC 12472 were collected from American Type Culture Collection (ATCC). Mueller–Hinton (MH) and Luria–Bertani (LB) media were prepared using distilled water. *P. aeruginosa* was subcultured into a fresh MH agar plate and incubated at 37 °C, while *C. violaceum* was subcultured into an LB agar plate and incubated at 30 °C for 24 h. Before each assay, the 24 h-old bacterial strains of *P. aeruginosa* and *C. violaceum* were re-cultured by inoculating single colonies in MH and LB broths and incubated with shaking maintained at 120 rpm at 37 °C and 30 °C, respectively. The overnight cultures were assessed for an OD600 nm of 0.8 to achieve 0.5 McFarland standard equivalent [17].

#### 2.2.2. Microdilution Assay for Antibacterial Activity

The minimum inhibitory concentration (MIC) of the oils and those of the reference standards (azithromycin and cinnamaldehyde) were determined using the broth dilution method [17,25]. Briefly, using 96-well microtiter plates (Merck Sigma-Aldrich, Johannesburg, South Africa), 100 µL of Mueller–Hinton broth (MHB) was transferred into each well, after which 200 µL two-fold serial dilutions of the antibacterial agents (oils (concentration ranging from 0.72 to 367.2 mg/mL) and positive controls (azithromycin and cinnamaldehyde (0.031–4 mg/mL)) and 2% DMSO (negative controls) was transferred into the wells. This was followed by the addition of 100 µL of standardized bacterial inoculum (1.33 × 10^8^ CFU/well), and each treatment was performed in triplicates. After 24 h of incubation at 37 °C, 40 µL of P–iodonitrotetrazolium (INT, 0.2 mg/mL) was added and incubated for another 45 min. Bacterial growth inhibition (clear wells with no color change) was observed and recorded visually. The MIC of the oils was recorded as the lowest concentration that inhibited bacterial growth with no visible growth [26].

#### 2.2.3. Evaluation of Oils for Anti-Quorum Sensing (AQS) Potential

##### Qualitative Anti-Quorum Sensing Assay

For this assay, a well diffusion method was performed according to Chenia [27] to detect the AQS activity of the oils. In brief, *C. violaceum* was grown in a sterile LB broth for 24 h at 30 °C, and the bacterial suspension was adjusted to an OD600 nm of 0.8. The standardized culture was evenly swabbed with a sterile swab across the sterile agar plate surface. The wells were inoculated with 100 µL of oils at varying concentrations of 91.8–11.48 mg/mL (MIC to 1/8 MIC) in triplicates, with reference standards of cinnamaldehyde at 3.75–0.47 mg/mL (MIC to 1/8 MIC) and azithromycin at 0.25–0.031 mg/mL (MIC to 1/8 MIC) (positive QS inhibitors) and negative control of 2% DMSO and incubated at 30 °C for 24 h. The plates were examined for violacein production, with the loss of purple violacein pigment (opaque zone) surrounding the wells indicating AQS and clear and transparent zones indicating bactericidal activity. As described by Cosa et al. [28], the diameter of the clear/halo inhibition zones was interpreted as follows: Susceptible (S) ≥ 15 mm, Intermediate (I) = 11–14 mm, and Resistant ≤ 10 mm.

##### Quantitative Anti-Quorum Sensing Assay

The oils were tested for the quantitative AQS activity at various concentrations ranging from MIC to 1/8 MIC against the *C. violaceum* using the microdilution method described by Pauw and Eloff [24]. Cinnamaldehyde (MIC: 3.75–1/8 MIC: 0.47 mg/mL) and azithromycin (MIC: 0.25–1/8 MIC: 0.031 mg/mL) served as reference standards. Before incubation, the absorbance was measured at OD600 nm (to check the bacterium’s viability and growth) and OD485 nm (violacein production). The 96-well plate was then incubated (120 rpm) at 30 °C for 24 h. After incubation, absorbance was measured again at OD420 nm. Subsequently, the plate was dried in a drying oven at 50 °C for 24 h. After drying, 150 µL of 100% DMSO was used to re-suspend the dried contents in each well, mixed thoroughly, and placed in the shaking incubator at 30 °C, 120 rpm for 1 h to confirm that the essential oils inhibited quorum sensing without affecting bacterial growth activities. Thereafter, absorbance was measured at an OD485 nm to determine the concentration of violacein. The analysis was performed in triplicates. Equation (1) was used to calculate the percentage (%) of inhibition [25].

(1) $$\mathrm{Percentage}\;\left(\%\right)\mathrm{inhibition}=\frac{\left(\mathrm{OD}\;\mathrm{control}-\mathrm{OD}\;\mathrm{test}\right)}{\mathrm{OD}\;\mathrm{control}}\times 100$$

Control: Controls (negative (*C. violaceum* treated with 2% DMSO), positive (cinnamaldehyde and azithromycin)), Test: Oil extracts (AGSUN 5102 CLP and AGSUN 5106 CLP).

#### 2.2.4. Inhibition of Cell Attachment

The oils and standards (azithromycin and cinnamaldehyde) were used to test for inhibition of cell attachment (anti-adhesion) against *P. aeruginosa*. The method used was slightly modified from that of Famuyide et al. [29]. A Sigma^®^ 96-well microtiter plate (Merck Sigma-Aldrich, Johannesburg, South Africa) was used where a 100 µL of standardized bacterial suspension (OD600 nm = 0.8), 100 µL of MHB, and 100 µL of oil were added to the wells at various concentrations (MIC to 1/8 MIC) in triplicates. The reference standards (azithromycin and cinnamaldehyde) and negative control (2% DMSO) were also added into their respective wells. The blank wells contained 200 µL of sterile MHB, and all the treatments were thereafter incubated at 37 °C for 24 h [17]. The modified crystal violet (CV) assay was used to assess biofilm biomass. To remove planktonic cells and media, the 96-well microtiter plates containing formed biofilm were washed three times with sterile distilled water. The plate was then oven-dried for 45 min at 60 °C. After drying, 1% CV solution was used to stain the remaining biofilm in the dark for 15 min. To remove any unabsorbed stains, the wells were washed three times with sterile distilled water. A semi-quantitative assessment of biofilm formation was performed by de-staining the wells with 125 µL of 95% ethanol. Subsequently, 100 µL of the de-staining solution was transferred to a new plate, and the absorbance (OD585 nm) was measured with a SpectraMax^®^ paradigm multimode microplate reader (Molecular Devices, Separations, South Africa) and the inhibitory effect of the oils and standards were calculated [17]. The following criterion for interpreting results was used: values between 0 and 100% were interpreted as inhibitory activity, and it was further broken down as follows: ≥50% (high activity) and values between 0 and 49% (low activity) [29].

#### 2.2.5. Inhibition of Biofilm Development

A total of 100 µL of standardized bacterial suspension and 100 µL of MHB were added to a 96-well microtitre plate (Merck Sigma-Aldrich, Johannesburg, South Africa) for biofilm development bioassays and incubated at 37 °C for 8 h. Thereafter, 100 µL of the oils and reference standards (azithromycin and cinnamaldehyde) were added to the wells at various concentrations (MIC to 1/8 MIC) in triplicates and incubated for another 24 h. Subsequently, the CV staining protocol was carried out as described in Section 2.2.4, the inhibitory effect of the test oils and standards was determined, and the results obtained were interpreted as previously reported [29].

#### 2.2.6. Confocal Laser Scanning Microscopy (CLSM)

Confocal laser scanning microscopy was used to evaluate the viability of biofilms, as earlier reported [30]. *Pseudomonas aeruginosa*’s biofilm was grown on glass pieces (1 × 1 cm) positioned in 24-well polystyrene incubated at 37 °C for 8 h. Following an 8-h incubation period, the preformed biofilm was supplemented with the MIC of the oils and standards (azithromycin and cinnamaldehyde) in triplicates and incubated for another 24 h. The adherent biofilm was delicately washed with deionized water before smearing with a live/dead backlight viability kit comprised of Syto 9 fluorescence and propidium iodide (PI) and incubated in the dark for 15 min. The plate was washed once more after staining. SYTO 9 fluorescence was identified using a Zeiss LSM 510 (Carl Zeiss Microscopy, Jena, Germany) confocal laser-scanning microscope with excitation at 488 nm, and the emission was collected with a 500–530 bandpass filter.

#### 2.2.7. Pyocyanin Assay

The pyocyanin assay was carried out using a slightly modified method of Bhattacharya et al. [31]. In a nutshell, an overnight *P. aeruginosa* culture was diluted to an OD600 nm of 0.8. Following that, the oils and standards (azithromycin and cinnamaldehyde) were added in various concentrations (MIC to 1/8 MIC) in triplicates, along with the standardized culture in King’s A broth, and incubated overnight at 37 °C. A 1.5 mL volume of overnight culture was centrifuged at 3000× *g* for 10 min. Thereafter, 1 mL of the supernatant was transferred into new centrifuge tubes (pre-cooled in ice), allowed to chill, and 100 µL chloroform was added while still on ice. Then, 300 µL of 0.2 M hydrochloric acid was added and vigorously mixed with a vortex mixer (EINS Sci E-VM-A, Biotechnology Hub Africa, Hatfield, South Africa). The pyocyanin-containing chloroform layer was collected and transferred to a Sigma^®^ 96-well microtiter plate (Merck Sigma-Aldrich, Johannesburg, South Africa). The absorbance was measured using a SpectraMax^®^ paradigm microtiter plate reader (Molecular Devices, Separations, South Africa) at 520 nm. To obtain the mean value, the experiments were repeated three times. The concentration of pyocyanin was calculated by multiplying the OD at 520 nm by 17.072 (the molar extinction coefficient). Pyocyanin production was compared to that of untreated cells, which was used as a control [17].

#### 2.2.8. Swarming and Swimming Motility Assays

The swarming assay medium consisted of nutrient broth (0.8%, *w*/*v*) supplemented with glucose (3%, *w*/*v*) and 0.5% (*w*/*v*) agar. The standardized bacterium, *P. aeruginosa* (2 µL), was spotted on swarming media supplemented with or without the oils on agar plates. Oils were tested at various concentrations (MIC to 1/8 MIC) in triplicates, with positive (azithromycin and cinnamaldehyde) and negative (2% DMSO) controls, respectively. The plates were incubated at 37 °C for 24 h, and thereafter, the zone diameters (mm) were measured and compared to the negative and reference standards to assess swarming motility. To obtain the mean value, the experiments were carried out in triplicate [17].

The swimming assay was carried out using swimming media as previously described [32] with minor modifications using 1% tryptone, 0.5% NaCl, and 0.5% agar. The plates were inoculated with 2 µL of *P. aeruginosa* (OD600 nm of 0.8) and either the oils or the controls (azithromycin and cinnamaldehyde) at various concentrations (MIC-1/8 MIC) in triplicates and incubated at 37 °C for 24 h. The turbid zone diameter (mm) was then measured by comparing the observation with the negative control (2% DMSO).

### 2.3. Chromatography

#### 2.3.1. Gas Chromatography-Mass Spectrophotometry (GC-MS) Analysis of the Oils

The chemical composition of the oils from the seeds (AGSUN 5102 CLP and AGSUN 5106 CLP) was determined using a GC-MS Shimadzu QP 2100 SE (Shimadzu Corporation, Tokyo, Japan) equipped with an Inert Cap 5 MS/NP capillary (30 m × 0.25 mm × 0.25 µm: GL Sciences, Tokyo, Japan) capillary column. The temperature of the column oven was initially held at 80 °C, then increased at a rate of 5 °C/min to 200 °C with a hold period of 2 min, and then increased to 280 °C with a hold time of 10°C/min. The temperature of the GC injector was set to 270 °C. A continuous flow rate of 1 mL/min of helium was used as a carrier gas. In full scan mode, electron ionization (EI) mass spectra were acquired throughout the range of m/z 40–550 at 70 eV ionization voltages. The temperatures of the ion source and transfer line were set to 200 and 250 °C, respectively. By comparing the retention times and mass spectra of the components to those of the National Institute of Standards and Technology (NIST) 05 mass spectral database, the components of the oils were identified [33].

#### 2.3.2. High-Performance Liquid Chromatography (HPLC) Analysis of the Oils for Phylloquinone Identification

The phylloquinone in the oils was identified using an HPLC system comprised of a D-6000 Merck Hitachi Interface, LC-Solution Software, an AS-4000A Intelligent Autosampler (Merck, Vienna, Austria) equipped with a Rheodyne 7125 injection valve and a 50 µL loop (Cotati, California), an L–6000 Merck Hitachi pump, and an L-7400 LaChrom UV Detector from Merck Hitachi. A LiChroCart^®^ RP-18 Lichrosphere^®^, 5 µm, 250–4.6 mm (Merck) column dry-packed with zinc powder was used, with a 4 × 4 mm guard column and RF-10AXL fluorescence detector. The extracted phylloquinone fraction from the oil sample was dissolved in 200 μL of mobile phase, and 50 μL was injected. The separation was in reverse phase with a LiChrospher RP-18 5 μm end-capped LiChroCART 250–4.6 column, with a pre-column from Merck and a mobile phase consisting of dichloromethane/methanol (10:90 *v*/*v*) with the addition of 5 mL of methanol solution with zinc chloride (1.37 g), sodium acetate (0.41 g), and acetic acid (0.30 g) per liter of mobile phase and was pumped at a flow rate 1.00 mL/min with isocratic elution. The post-column reduction (20 × 4.0 mm id) was filled manually with zinc dust p.a. grade from Merck with particles < 45 μ and kept in a furnace (Shimadzu-CTO-6A) at 40 °C with fluorescence detector excitation 243 nm and emission 430 nm. Phylloquinone peaks were identified by comparing their retention times to those of standards. Concentrations were calculated from peak areas determined using a Jasco ChromNav Chromatography Data System (Jasco Corporation) [34].

### 2.4. Computational Analyses

#### 2.4.1. Molecular Docking

##### Proteins Retrieval, Prepping, and Active Site Identification

The X-ray crystal structures of the Las protein from *P. aeruginosa* (LasR: 2UV0, LasA: 3IX7, LasB: 3DBK, LasI: 1RO5, ToxA: IXK9, AprA: 1KAP) and the CviR QS protein from *C. violaceum* (CviR: 3QP1, VioA: 6G2P) were downloaded from the RCSB Protein Data Bank (https://www/rcsb.org/pdb) (accessed on 16 May 2022). The proteins were saved in pdb format using the UCSF Chimera 1.15 software package after optimization via the removal of all non-standard residues, non-essential water molecules, and other heteroatoms (co-crystallized ligands) attached to the protein. The active site coordinates were identified via Discovery Studio 2021. They were validated by optimally superimposing docked ligands against the reference pocket containing the native ligand in the experimental co-crystallized LasR structure. The Root Mean Square Deviation (RMSD) value (0.5 Å) obtained between the docked ligands and the native inhibitor orientation confirmed the approach used [19,20,21,22,23].

##### Ligand Procurement

The standard drugs (azithromycin and cinnamaldehyde) and all phytochemical compounds identified from GC-MS were acquired in sdf format from PubChem (www.pubchem.com) (accessed on 16 May 2022).

##### Docking

Subsequent to the optimization of the proteins and ligands, the python prescription (PyRx-0.8) software coupled with AutoDock vina was used for docking. The phytoconstituents of the oils and standard (azithromycin and cinnamaldehyde) were docked into the active sites of each of the Las proteins (*P. aeruginosa*) and CviR QS proteins (*C. violaceum*) by selecting the amino residues present in a 3-D structure of a co-crystallized protein downloaded from the protein data bank. The best binding pose with the highest binding affinity was saved in pdb format, and each final complex was visualized in Discovery Studio 2021 for the interactions formed between the ligands and Las protein and CviR. The standards and the phytoconstituents with the best interaction and docking scores against the most vulnerable Las protein (Las protein in which the phytoconstituents had best docking scores) from *P. aeruginosa* and the most vulnerable CviR protein from *C. violaceum* were then subjected to MD simulation [35].

#### 2.4.2. Molecular Dynamics Simulation

The MD simulation was carried out on the Center for High Performance and Computing’s system, using AMBER 18 software to run the FF18SD variation of the amber force field. The TIP3P water molecules, Na+, and Cl^−^ counter ions were included to neutralize the system using the ANTECHAMBER atomic partial charges assigned to the ligands, and the non-bond interactions cut-off value was adjusted to 8 Å using the ANTECHAMBER atomic partial charges assigned to the ligands. The system started with 2000 minimization steps, was constrained with 500 kcal/mol potential for both solutes on another 1000 steps with the help of the steepest descent approach, and was then followed by conjugate gradients of 1000 steps. Then, using the conjugate gradient technique, the entire minimization stage was carried out in 1000 unrestricted steps. To maintain a constant volume of water and atoms, the following MDS stage involved 50 ps of heating from absolute zero (0 K) to 300 K. The solutes were then exposed to collision frequency and possible harmonic constraint (10 kcal/mol) (1.0 ps). The system was then brought to equilibrium by applying 500 ps while maintaining a constant heating temperature of 300 K. Other factors, such as several atoms and pressure (at 1 bar), were kept constant to simulate an isobaric–isothermal ensemble (NPT) [35]. While each MD simulation took 100 ns on average and contracting H^+^ by simulation using the SHAKE algorithm using the SPFP precision model, the step size was reduced for the bond 2 fs. NPT, constant pressure, and the simulations agreed [35]. Temperature (300 K), pressure coupling constant (2 ps), randomized seeding, and a Langevin thermostat number of collisions (1.0 ps) [35]. The CPPTRAJ program was used to process the trajectories for the analyses of the post-MD simulation parameters such as root–mean square deviation (RMSD), root–mean square fluctuation (RMSF), and radius of gyration (RoG), while the Leap Module Shake algorithm was used to reduce the expansion of chemical bonds involving hydrogen atoms. The molecular Mechanics/GB Surface Area technique (MM/GBSA) was utilized to compute the binding free energy for each ligand–protein combination over a period of 100 ns simulation [36]. Based on Ylilauri and Pentikäinen [37], the free binding energy (G) was calculated using the Molecular Mechanics/GB Surface Area Method (MM/ GBSA). This was performed to estimate the systems’ binding affinities. In a 100-ns trajectory, the average of G across 100,000 photos was found.

#### 2.4.3. Pharmacokinetic Properties

The SwissADME server (http://www.swissadme.ch/) (accessed on 21 July 2022) was used to estimate the pharmacokinetic parameters (absorption, distribution, metabolism, and excretion (ADME) features and drug-likeness) of the top three compounds of the oils and the controls.

### 2.5. Statistical Analysis

Except otherwise stated, the in silico and in vitro results were expressed as the mean ± standard error of the mean (SEM) and in percentages (%), and statistical analyses were carried out using the Graph Pad Prism version 5.0 and one-way analysis of variance (non-parametric tests) to determine the significant difference (*p* < 0.05) between the treatment means.

## 3. Results

### 3.1. In Vitro Analyses

#### 3.1.1. Minimum Inhibitory Concentration

A color change was observed after adding INT. Pink coloration indicated no inhibition, while clear zones indicated significant inhibition. This was performed to determine the MICs of the oils against planktonic growth of *P. aeruginosa* ATCC 27853, and the MICs obtained for both oils were 91.80 mg/mL each, while those of azithromycin and cinnamaldehyde were 0.25 mg/mL and 3.75 mg/mL, respectively (Table 1). The negative control, 2% DMSO, showed no inhibitory effect (Table 1).

**Figure 1:**
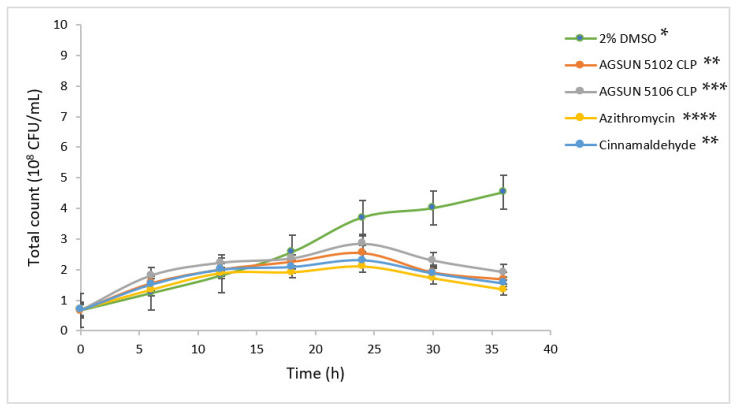
Growth curves of *P. aeruginosa* supplemented with different treatments at 37 °C for 36 h. The growth profiles are statistical difference at *p*-value ≤ 0.05 with *, **, *** and **** denoting significant differences between the treatments.

**Figure 2:**
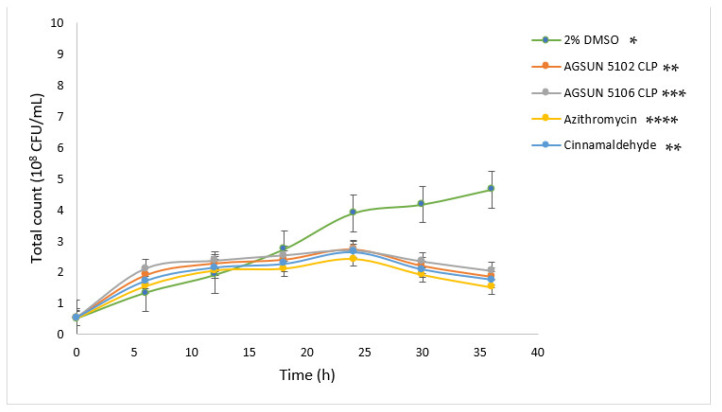
Growth curves of *C. violaceum* supplemented with different treatments at 37 °C for 36 h. The growth profiles are statistical difference at *p*-value ≤ 0.05 with *, **, *** and **** denoting significant differences between the treatments.

An exponential growth was seen in *P. aeruginosa* (Figure 1) and *C. violaceum* (Figure 2) treated with 2% DMSO throughout the incubation period, with a total bacterial count of 4.53 × 10^8^ CFU/mL and 4.65 × 10^8^ CFU/mL, respectively, after 36 h. However, while the lag phase (before 15 h for both organisms) of the oil- and reference standards-treated cells was comparable to the negative control (2% DMSO-treated cells), a moderate decrease in bacterial counts was observed for both organisms after 15 h, with the most profound effect observed with azithromycin (1.1 × 10^8^ CFU/mL for both organisms) after 36 h. The total bacterial count observed for AGSUN 5102 CLP-treated cells was comparable to the cells treated with cinnamaldehyde, which were significantly different from cells treated with AGSUN 5106 CLP at *p* ≤ 0.05 for both organisms (Figure 1 and Figure 2).

#### 3.1.2. Anti-Quorum Sensing (Qualitative and Quantitative)

**Figure 3:**
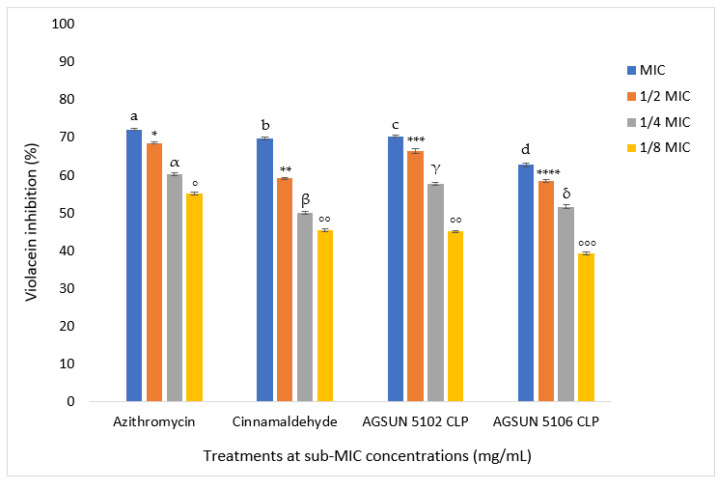
Inhibitory effect of the oils on the production of violacein by *C. violaceum* ATCC 12472 at sub-MIC concentrations (mg/mL) of azithromycin at 0.25–0.031 mg/mL (MIC to 1/8 MIC), cinnamaldehyde at 3.75–0.47 mg/mL (MIC to 1/8 MIC) and both oils (AGSUN 5102 CLP and AGSUN 5106 CLP) at 91.8–11.48 mg/mL (MIC to 1/8 MIC). Letters/symbols represent statistical differences at *p*-value ≤ 0.05.

Treatments with the oils caused loss of the purple violacein pigment of *C. violaceum* that QS mediates (opaque halo) around the agar wells following the addition of essential oils with zones of between 1.00 and 2.33 mm in diameter at 91.80 mg/mL (MIC) with AGSUN 5102 CLP having the highest zone of inhibition (Table 2 and Figure S1d,e from Supplementary Materials). On the other hand, azithromycin and cinnamaldehyde elicited AQS activity with distinct zones of inhibition of 13.33 mm and 5.33 mm at 0.25 mg/mL and 3.75 mg/mL (MIC), respectively. However, 2% DMSO (negative control) showed no discernible zones of inhibition (Table 2 and Figure S1a–c from Supplementary Materials). Moreover, at concentrations lesser than the MIC, no zones of inhibition were observed. However, a concentration-dependent AQS activity was quantitatively observed after treatment with essential oils and reference standards. At MIC, AGSUN 5102 CLP and AGSUN 5106 CLP showed 72.38% and 62.96% inhibition, respectively, while cinnamaldehyde and azithromycin showed 74.23% and 70.42% inhibition, respectively (Figure 3).

#### 3.1.3. Inhibition of Cell Attachment and Biofilm Formation

**Figure 4:**
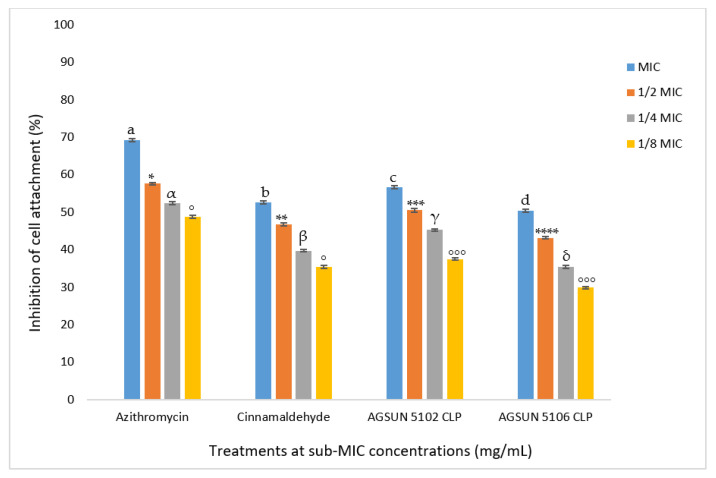
Inhibitory effect of the essential oils on cell attachment of *P. aeruginosa* ATCC 27853 at sub-MIC concentrations (mg/mL) of azithromycin at 0.25–0.031 mg/mL (MIC to 1/8 MIC), cinnamaldehyde at 3.75–0.47 mg/mL (MIC to 1/8 MIC), and both oils (AGSUN 5102 CLP and AGSUN 5106 CLP) at 91.8–11.48 mg/mL (MIC to 1/8 MIC). Letters/symbols represent statistical differences at *p*-value ≤ 0.05.

**Figure 5:**
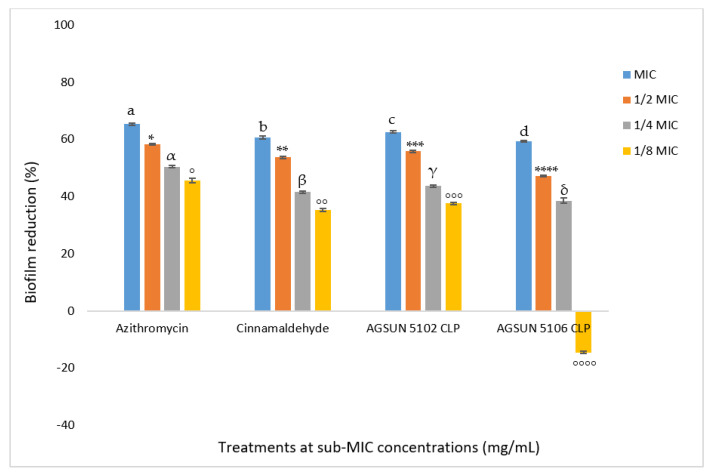
Inhibitory effect of the essential oils on biofilm formation of *P. aeruginosa* ATCC 27853 at sub-MIC concentrations (mg/mL) of azithromycin at 0.25–0.031 mg/mL (MIC to 1/8 MIC), cinnamaldehyde at 3.75–0.47 mg/mL (MIC to 1/8 MIC), and both oils (AGSUN 5102 CLP and AGSUN 5106 CLP) at 91.8–11.48 mg/mL (MIC to 1/8 MIC). Letters/symbols represent statistical differences at *p*-value ≤ 0.05.

The oils showed favorable competition with the reference standards for biofilm and cell attachment inhibition. It was observed that the inhibitory effect of the oils on *P. aeruginosa* biofilm formation and cell attachment was concentration-dependent (Figure 4 and Figure 5). At MIC, AGSUN 5102 CLP and AGSUN 5106 CLP had 56.64% and 50.34% inhibitory effect on cell attachment, respectively, while azithromycin and cinnamaldehyde elicited 70.24% and 55.42% inhibitory effect on cell attachment, respectively (Figure 4). Similarly, treatment with AGSUN 5102 CLP and AGSUN 5106 CLP at MIC showed that biofilm formation was reduced by 62% and 59%, respectively, while azithromycin and cinnamaldehyde showed 65% and 60% inhibitory effect, respectively. However, an improvement in biofilm formation (−14.52%) was observed after treatment with AGSUN 5106 CLP at 1/8 MIC (Figure 5).

#### 3.1.4. Confocal Laser Scanning Microscopy

**Figure 6:**
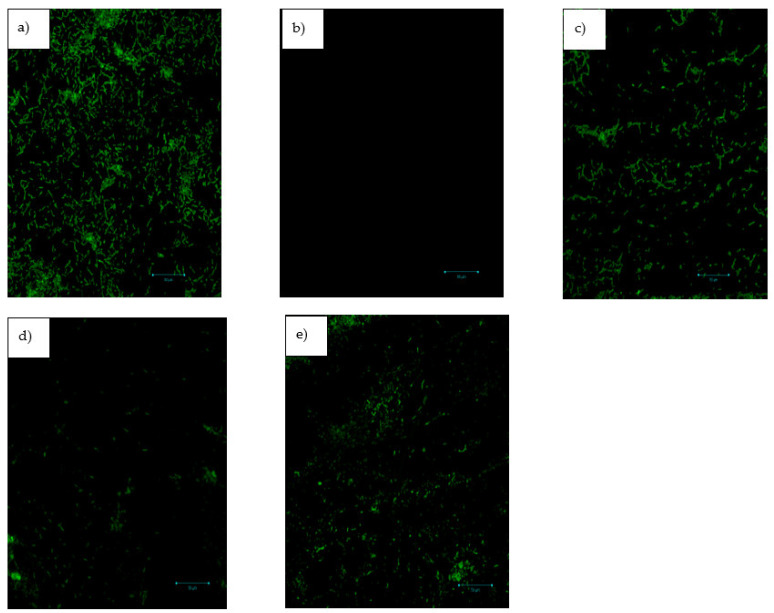
Confocal laser scanning micrograph (×500) of (**a**) 2% DMSO, (**b**) azithromycin, (**c**) cinnamaldehyde, (**d**) AGSUN 5102 CLP, and (**e**) AGSUN 5106 CLP.

Upon treatment of *P. aeruginosa* cells with the oils, a significant reduction in biofilm matrix, thickness, and biomass was observed (Figure 6). In the untreated cells or those treated with negative control (2% DMSO), dominant biomass and thickness of biofilms were distinctly observed (Figure 6a). However, there was a significant reduction upon supplementation with the oils. Treatment with azithromycin completely inhibited biofilm matrix formation (Figure 6b), whereas the effect produced by treatment with cinnamaldehyde (Figure 6c) was comparable with those of the oils (Figure 6d,e), with the effect observed with AGSUN 5102 CLP being more pronounced in reducing the thickness and biomass of biofilms (Figure 6d).

#### 3.1.5. Inhibition of Pyocyanin

**Figure 7:**
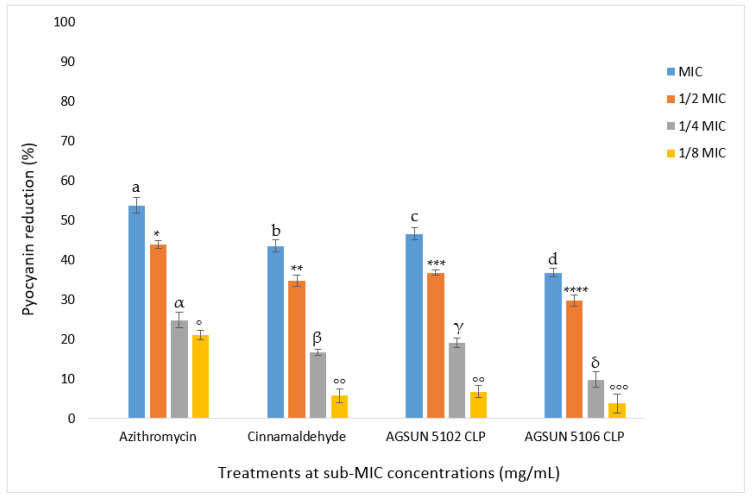
Inhibitory effect of essential oils on QS-mediated virulence factor pyocyanin on *P. aeruginosa* ATCC 27853 at sub-MIC concentrations (mg/mL) of azithromycin at 0.25–0.031 mg/mL (MIC to 1/8 MIC), cinnamaldehyde at 3.75–0.47 mg/mL (MIC to 1/8 MIC), and both oils (AGSUN 5102 CLP and AGSUN 5106 CLP) at 91.8–11.48 mg/mL (MIC to 1/8 MIC). Letters/symbols represent statistical differences at *p*-value ≤ 0.05.

A concentration-dependent reduction of pyocyanin was observed following treatment with the oils and reference standards. Pyocyanin production was significantly inhibited (*p*-value ≤ 0.05) following treatment (at MIC), with AGSUN 5102 CLP (46.46%) and AGSUN 5106 CLP (31.81%) competing well with azithromycin (53.81%) and cinnamaldehyde (43.60%) (Figure 7).

#### 3.1.6. Inhibition of Swarming and Swimming Motility

Treatment with the oils significantly (*p* ≤ 0.05) decreased swarming motility in the cells compared to the untreated control (Table 3 and Figure S2 from Supplementary Materials) at different doses. The highest inhibition was observed at the MIC (91.80 mg/mL), with AGSUN 5102 CLP (2.0 mm) and AGSUN 5106 CLP (3.0 mm) having activity comparable to the reference standards azithromycin (2.0 mm) and cinnamaldehyde (2.5 mm) (Table 3).

As shown in Table 4, treatment with the oils and standards decreased swimming motility at all the investigated concentrations. Most of the reduction was observed at the highest concentrations (MIC) of the oils and reference standards (Table 4 and Figure S3 from Supplementary Materials). Compared to the untreated cells (34.0 mm), treatment with the oils (at MIC) significantly (*p* ≤ 0.05) reduced motility with zone diameters of 21.0 mm (AGSUN 5102 CLP) and 23.0 mm (AGSUN 5106 CLP) (Figure S3d,e from Supplementary Materials), which was higher than that observed for azithromycin (17.0 mm) but comparable to 22.0 mm observed for cinnamaldehyde (Table 4; Figure S3b,c from Supplementary Materials).

#### 3.1.7. Chromatography (GCMS and HPLC) Analysis of the Oil

**Figure 8:**
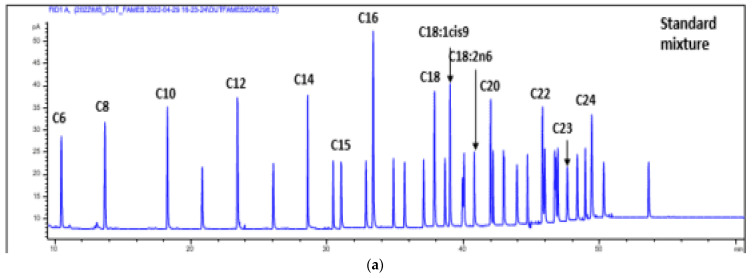

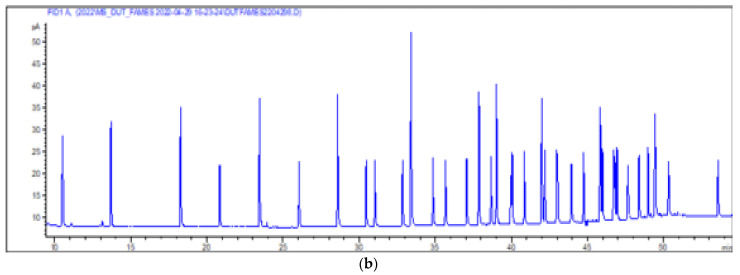
Representative total ion chromatograph (TIC) of (**a**) AGSUN 5102 CLP and (**b**) AGSUN 5106 CLP essential oils.

The results of the chromatographic analysis of oils revealed 15 similar constituents between the two cultivars; however, they differed in quantity and abundance (Table 5 and Figure 8a,b). The components identified were mainly fatty acids and vitamins, with linoleic and oleic acids being the most abundant components (Table 5). The full details of the mass-to-charge/ion (m/z) ratios and retention times of the compounds are presented in Table S1.

### 3.2. Computational Analyses

#### 3.2.1. Molecular Docking

**Figure 9:**
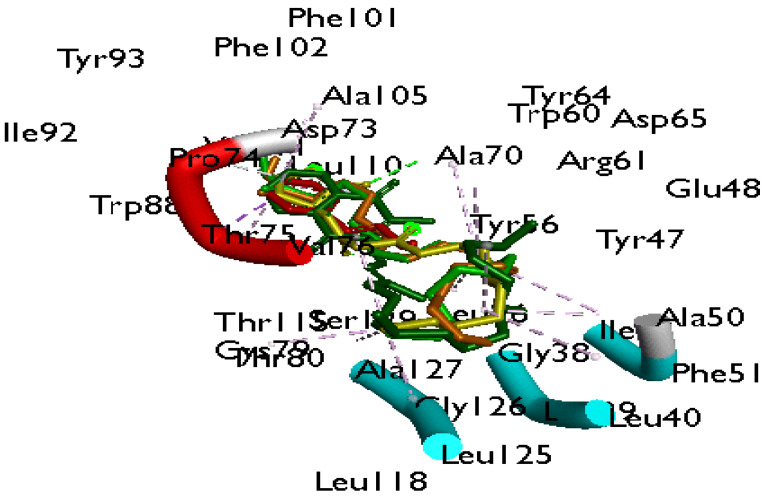
Superimposition of docked compounds on co-crystal structure of LasR (RMSD: 0.5 Å): Red: native inhibitor, Brown: standard drug (Cinnamaldehyde), Yellow: standard drug (Azithromycin), and Green: ligand with the highest binding affinity (Phylloquinone).

The docking scores of the 15 constituents from the two oils against Las proteins from *P. aeruginosa* and CviR from *C. violaceum* are shown in Table S2 from Supplementary Materials. Of the Las proteins, the constituents had higher negative docking scores (Table S3 from Supplementary Materials) and interactions (Table S4 from Supplementary Materials) against *P. aeruginosa* LasR. Phylloquinone (−9.4 kcal/mol), linoleic acid (−8.5 kcal/mol), and oleic acid (−8.2 kcal/mol) were the three most promising constituents/metabolites against LasR, judging by their higher interactions and docking scores relative to the reference standards (Azithromycin (−6.4 kcal/mol); cinnamaldehyde (−7.4 kcal/mol)), with phylloquinone being the most promising metabolite (Table 6). Similarly, of the two proteins (CviR and VioA) involved in the CviR QS system, the CviR protein was the most inhibited (Table S5 from Supplementary Materials). The top four metabolites (phylloquinone (−8.6 kcal/mol), linoleic acid (−6.7 kcal/mol), myristic acid (−6.6 kcal/mol), lauric acid (−6.6 kcal/mol)) of the oils against CviR had competitive docking scores (Table S6 from Supplementary Materials) and higher binding interactions (Table S7 from Supplementary Materials) compared to the reference standards (cinnamaldehyde (−7.1 kcal/mol), azithromycin (−5.3 Kcal/mol)), with phylloquinone having the highest docking scores and interactions. The data obtained regarding the validation of the docking protocol with the top three metabolites and the reference standards by optimal superimposition with the native LasR inhibitor yielded an RMSD value of 0.5 Å (Figure 9).

#### 3.2.2. Molecular Dynamics Simulation

**Figure 10:**
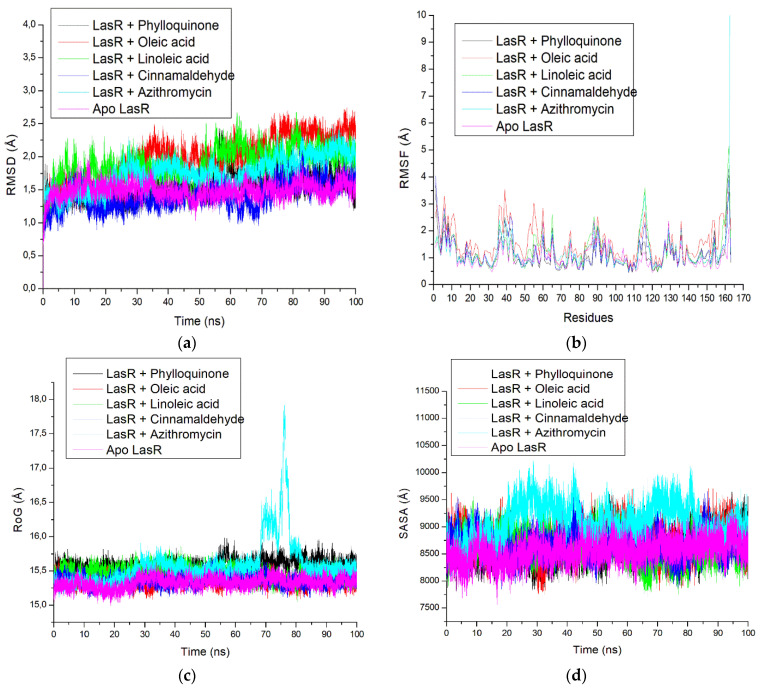

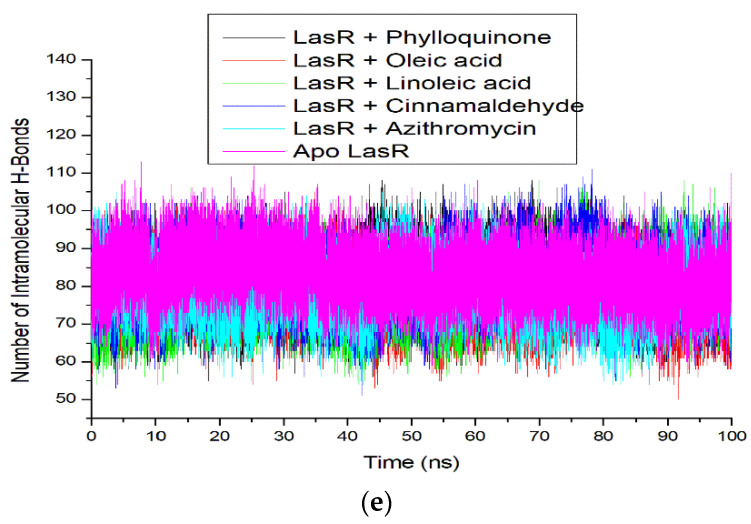
(**a**) Root mean square deviation (RMSD), (**b**) root mean square fluctuations (RMSF), (**c**) radius of gyration (RoG), (**d**) solvent accessible surface area (SASA), and (**e**) number of intramolecular hydrogen bonds plots of comparison between LasR and top three compounds, azithromycin, and cinnamaldehyde determined over 100 ns molecular dynamics simulation.

Compared to the standards, the top three compounds had significantly higher total binding free energy values ((−66.42 ± 4.63 kcal/mol (phylloquinone), −53.14 ± 3.53 kcal/mol (linoleic acid), and −52.02 ± 3.91 kcal/mol (oleic acid)) against LasR (Table 7) with phylloquinone having the highest value. The RMSD of the LasR–ligand complexes converged at approximately 15 ns and 45 ns, however after 60 ns, they were substantially stable (Figure 10a), with the least fluctuations at amino residues 15–35, 45–50, and 100–110 (Figure 10b). Lesser fluctuations in RoG (Figure 10c) and SASA (Figure 10d) were seen in LasR + cinnamaldehyde, LasR + phylloquinone, LasR + linoleic acid, and LasR + oleic acid complexes throughout the 100 ns simulation compared to LasR + azithromycin, with pronounced fluctuation in RoG between 70 and 90 ns and SASA between 20 and 85 ns (Figure 10c,d). Figure 10e shows a consistent fluctuation in the pattern of the number of hydrogen bonds produced in LasR, typically between 60 and 100 before and after the binding of the top three compounds of the oils, azithromycin, and cinnamaldehyde. Of the top three metabolites studied, phylloquinone had the lowest average RMSD (1.51 Å), RMSF (1.11 Å), and SASA (8623.64 Å) values compared to the values for azithromycin–LasR. LasR ligand binding generally increases mean RMSD, RMSF, and SASA values but has little effect on mean RoG values, with a negligibly small variance of 0.26 Å between the resulting complexes (Table 8). The top three compounds, alongside azithromycin and cinnamaldehyde, had different natures, bond lengths, and numbers of interactions at the active site of LasR after the 100 ns simulation, which impacted the resulting binding free energy values in this study (Table S10). LasR–cinnamaldehyde complex had a total of 13 bonds, with conventional hydrogen bonds (Trp54), π–π stacked and π–π T-shaped (Trp88 and Phe95), π–alkyl (Ala99), and van der Waals (Tyr58; Asp67; Thr69; Ile86, Pro60; Tyr87; Phe96; Leu104; Tyr50). The complex with azithromycin had 13 bonds, comprising conventional hydrogen bonds (Glu133), carbon–hydrogen bonds (Asp23; Leu24; Gly25; Phe26), unfavorable donor–donor (Arg136), alkyl (Val126 and Val141), and van der Waals (Ser27; Phe137; Ser22; Glu127; Ala128). LasR–phylloquinone complex, on the other hand, had 30 interactions, including conventional hydrogen bonds (Thr109 and Trp54), π–anion (Asp67), π–π stacked (Tyr58 and Trp82), alkyl and π–alkyl (Leu30; Ala121; Leu119; Ile46; Tyr41; Leu34; Ala44; Val70; Cys73; Leu104), and van der Waals (Asp59; Phe45; Gly32; Phe31; Gly20; Thr74; Ala64; Leu33; Thr69; Tyr87; Ser123; Arg55; Ala99; Phe95; Tyr50), when compared to LasR–linoleic acid complex with 23 interactions comprising conventional hydrogen bonds (Tyr87 and Leu104), alkyl and π–alkyl (Arg55; Tyr58; Val70; Trp54; Trp82; Ile46; Tyr41; Ala121; Leu30; Phe95), and van der Waals (Leu122; Gly32; Phe31; Asp67; Ala99; Val105; Phe96; Thr69; Thr109; Tyr50; Ser123). The oleic acid complex formed 29 interactions, including conventional hydrogen bonds (Glu83), carbon–hydrogen bonds (Trp82), alkyl and π–alkyl (Tyr87; Ala99; Trp54; Val70; Tyr58; Ala121; Leu30; Leu104), and van der Waals (Ile86; Pro84; Phe96; Phe95; Tyr50; Ser123; Ala64; Gly120; Tyr41; Leu34; Ala44; Leu33; Gly32; Phe45; Ile46; Phe31; Thr109 Asp67; Thr69) (Table S10). Against CviR of *C. violaceum*, higher binding free energy values were observed with the top four metabolites relative to the cinnamaldehyde (−16.90 kcal/mol), while in contrast to azithromycin (−32.05 kcal/mol), only phylloquinone (−50.56 kcal/mol) and linoleic acid (−41.17) had higher values, with phylloquinone having the highest value (Table S8 from Supplementary Materials). Interestingly, except for linoleic acid, the binding of CviR caused reduced mean RMSD values, with myristic acid (1.23 Å) having the lowest value (Table S9, Figure S4 from Supplementary Materials). Similarly, with the exception of linoleic acid and azithromycin, ligand binding of CviR caused reduced average RMSF values, with myristic acid (1.04 Å) having the lowest value (Table S9 from Supplementary Materials). The RoG, SASA, and intramolecular hydrogen bonds showed slightly increased values following binding of CviR, with azithromycin–CviR having the highest RoG and SASA values, while myristic acid–CviR had the highest intramolecular hydrogen bonds (Table S9, Figure S4 from Supplementary Materials). The top-ranked metabolites, azithromycin, and cinnamaldehyde all had different natures, bond lengths, and interactions at the CviR active site, which impacted the binding free energy values observed with the target (Figure S10 from Supplementary Materials).

**Figure 11:**
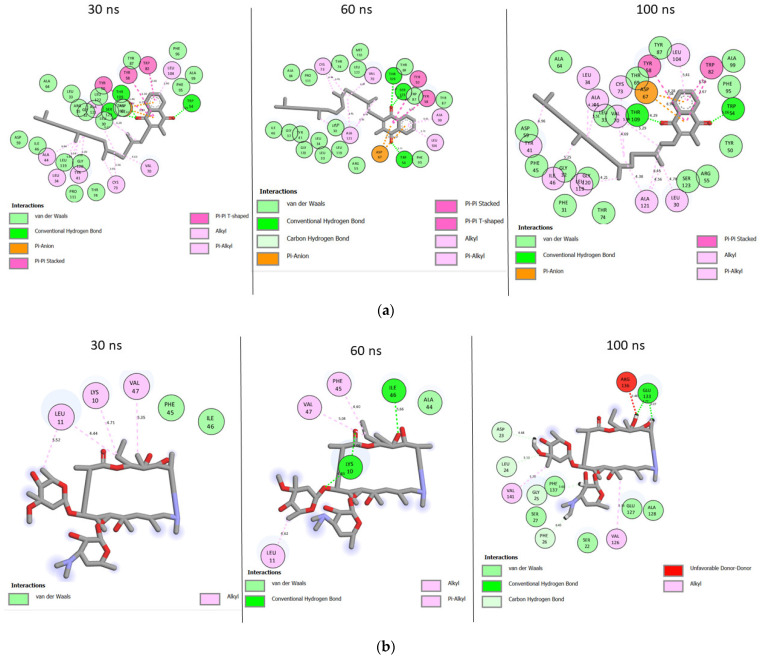
Plots of interactions at different intervals of (**a**) phylloquinone and (**b**) azithromycin against LasR during the 100 ns MD simulation.

During the simulation, a consistent number of interactions at 30 ns, 60 ns, and 100 ns were observed with the top-ranked metabolites, and reference standards against LasR and CviR (Table S10 from Supplementary Materials) and those formed between the metabolite (phylloquinone) (Figure 11a) and reference standard (azithromycin) (Figure 11b) with the highest affinity with LasR are presented in Figure 11. Phylloquinone–LasR complex had a total of 30, 29, and 30 interactions at 30 ns, 60 ns, and 100 ns, respectively (Figure 11a), while azithromycin–LasR complex formed 5, 6, and 13 interactions at 30 ns, 60 ns, and 100 ns, respectively (Figure 11b). Interactions formed between other top-ranked metabolites with LasR and those formed with CviR at 30 ns, 60 ns, and 100 ns are presented in Table S10.

#### 3.2.3. Pharmacokinetic Properties

The identified top three compounds against LasR in this study did fairly well with the violation of Lipinski’s rule of five, having a molecular weight of less than 500 g/mol (Phylloquinone (450.70 g/mol); Linoleic acid (280.447 g/mol); Oleic acid (282.470 g/mol)), hydrogen bond donors of less than 5 ((Phylloquinone (0); Linoleic acid (1); Oleic acid (1)), and hydrogen bond acceptors of less than 10 ((Phylloquinone (2); Linoleic acid (2); Oleic acid (2)) (Table 9). Azithromycin, on the other hand, had three violations (Table 9). While azithromycin and phylloquinone had a low gastrointestinal tract (GIT) absorption rate, linoleic acid and oleic acid had higher GIT absorption rates (Table 9). Azithromycin had the lowest bioavailability score, while phylloquinone had a bioavailability score that was comparable to that of cinnamaldehyde. Except for phylloquinone and azithromycin, which were predicted to be poorly soluble, the other top-ranked metabolites are moderately soluble in an aqueous environment (Table 9).

## 4. Discussion

There have been numerous studies conducted on sunflower seeds to determine their antibacterial, anticancer, antioxidant, and other health benefits [38,39,40]. However, no information exists on their anti-QS potential to date, hence the motivation for this study. Solvent extraction is the traditional method for obtaining oil from oilseeds, and in this study, n-hexane was employed as a solvent for extraction because of its benefits, including easy recovery, low latent heat of vaporization (330 kJ/kg), non-polarity, and good selectivity to solvents [41].

The MIC of an antibiotic is the lowest concentration at which bacterial growth is prevented [42]. In this study, the two oils (AGSUN 5102 CLP and AGSUN 5106 CLP) had remarkable MIC, which was higher than those obtained for the reference standards (azithromycin and cinnamaldehyde), and the one reported by Liu et al. [38] but were significantly lower than the values reported by Benites et al. [43]. Interestingly, despite the fact that the oils were not refined as the standards used in this study, the values obtained with the two cultivars fall within the limit reported by Ács et al. [44], which is >43.4 mg/mL. Thus, it can be inferred that *P. aeruginosa* showed considerable susceptibility to the active constituents of the oils from the two investigated cultivars of sunflower seeds. Hence, both oils could be further explored as possible antibacterial agents against the test pathogen in this study.

The pathogens were treated with the oils and reference standards for 36 h to understand the growth pattern of *P. aeruginosa* and *C. violaceum*. The initial steady growth observed before 15 h for the oils and reference standard-treated cells could be due to the low permeability of the outer membrane of Gram-negative bacteria, which enables them to express or exhibit innate resistance [45]. However, the observed decrease in bacterial growth beyond 15 h in cells treated with the oils and the reference standards could be attributed to the minute penetration of the outer membrane by the administered treatments, which could have initiated a slow release of bacterial cell components that eventually led to cumulative cell death. Ideally, for the oils to act as AQS or potential antipathogenic agents, they only need to show less hindrance to bacterial development, as the speculation of antipathogenic drugs ought not to be bactericidal but to hinder or disrupt the virulence factors [45].

*Chromobacterium violaceum* ATCC 12472, a well–recognized QSI biomonitor strain that generates the purple pigment known as violacein, was chosen as the QSI biomonitor strain as it is an efficient bacterium to visually detect and quantify pigment suppression by metabolites of the investigated oils in this study. Generally, violacein pigmentation in *C. violaceum* ATCC 12472, regulated by QS chemical communications, produces a naturally occurring and easily observable phenotype without the need for additional substrates, making it simple to assess how well a substance inhibits QS [46]. However, it is worth noting that this screening technique does not provide information on the precise kinds and quantities of active chemical compounds that are present [47]. In this study, treatment with the oils might have impacted violacein pigment, which could indicate that the pathogen’s QS system had been tampered with, as seen by the sizes of the opaque zones surrounding the wells administered with the oils. However, the small size of the opaque zones following treatments with the oils could indicate that the biomonitor strain exhibited resistant characteristics to the oils, which according to Kowalska–Krochmal and Dudek–Wicher [42], suggest a substantial possibility of therapeutic failure even with increasing doses of the oils. On the other hand, azithromycin, which is a refined drug, demonstrated an intermediate characteristic at MIC towards the biomonitor strain, which was largely anticipated, suggesting a likelihood of therapeutic success by increasing the dosage of the medication. Generally, judging by the results obtained, it could be logically inferred that qualitative violacein inhibitory assay might not be the best method to explore AQS potentials as a therapeutic agent, and this was consistent with the submission of Cosa et al. [28]. Hence, the need for quantitative studies to complement qualitative assays in evaluating the AQS potential of an agent.

In this study, to further validate the interference of the QSS of *C. violaceum* by the oils through inhibition of the purple pigment as suggested by Cosa et al. [28], a quantitative AQS was performed. The two oils exhibited varying levels of AQS activity against *C. violaceum,* as confirmed by the significantly reduced production of violacein following treatment by AGSUN 5102 CLP and AGSUN 5106 CLP at both MIC and sub-MIC doses. This observation agrees with a previous study that demonstrated that antipathogenic medications should show their AQS potential with their effectiveness shown at sub-MIC concentrations and upward [45]. In a related investigation, Khan et al. [48] noted a remarkable halt in the formation of the violacein pigment in *C. violaceum* when essential oils of cinnamon, peppermint, and lavender were present. Similarly, according to Noumi et al. [49], tea tree oil demonstrated significant inhibition of violacein in *C. violaceum* ATCC 12472 at MIC and sub-MIC. In this study, like the essential oils, both cinnamaldehyde and azithromycin also had significant AQS activity at all doses. These findings are significant, as they demonstrate that some metabolites of the oils had a structural resemblance to the analogs of signaling molecules (AHL), allowing competitive structural binding between AHL and the oils’ metabolites with the appropriate receptor protein in the organism, thus, affecting the transmission of signal molecules and QS due to essential oils’ metabolites interfering with the *C. violaceum* CviR-QSS. The results of this study showed that the oils could prevent the development of QS.

The current study also aimed to investigate the ability of the oils to get rid of biofilms of *P. aeruginosa* both in early and mature stages. The oils significantly inhibited cell attachment in a concentration-dependent manner, coherent with what was seen in the quantitative AQS. Both oils favorably competed with the standards in reducing cell attachment. The anti-adhesion activity observed with the oils in this study agrees with the findings of Pejčić et al. [50], who observed that the presence of sage and basil essential oils significantly inhibited cell attachment. Since cell attachment is significant in the advancement of infection caused by *P. aeruginosa* [17], their inhibition during the early stages of biofilm formation by the essential oil suggested their importance as an AQS antimicrobial. As in the inhibition of cell attachment, a similar trend in the inhibition of biofilm development was observed in this study, where both oils competed relatively well with the controls in reducing biofilm formation. Remarkably, the reduction of biofilm formation by both oils was observed at all concentrations except at 18MIC of AGSUN 5106 CLP, where the biofilm formation was enhanced. This observation points to the AQS potency of the essential oils. This phenomenon has also been reported by other researchers [17,51]. However, one explanation for biofilm formation enhancement at 18MIC of AGSUN 5106 CLP could be due to the efflux pump resistance mechanism during the cell attachment stage, where AGSUN 5106 CLP oil might have been expelled from the cells [17]. Another possible reason for biofilm formation enhancement at 18MIC could be because some of the metabolites of the oil were utilized by the bacteria as a source of nourishment and aided in the growth of the bacteria.

Examining biofilm structure in relation to the geographic localization of significant biofilm matrix components is possible using microscopy techniques. Confocal laser scanning microscopy (CLSM) is an effective tool for studying biofilms and may be used to quantitatively examine the biofilm matrix and the amount of adhering biomass [52]. Additionally, the spatiotemporal impacts of various nutritional conditions or antibiotic treatments can be observed [53]. In this study, CLSM was used to study the three-dimensional architectural complexity of biofilms in the presence of the oils. The marked reduction in biofilm thickness and biomass following treatment with the oils compared to the 2% DMSO-treated cells could be indicative of the anti-biofilm activity of the oils. Compared to the reference standards, a complete inhibition of biofilm thickness and biomass was observed in the azithromycin-treated cells. This finding corroborates the cell attachment and biofilm formation inhibition assay, as azithromycin was the most effective anti-biofilm agent. However, treatment with the oils, especially AGSUN 5102 CLP, showed activities comparable to cinnamaldehyde-treated cells, where the biofilm thickness and biomass were significantly reduced, which also corroborates the findings of the cell attachment and biofilm inhibition assay. This is a significant finding as it shows that the oils had the ability to turn off the expression of genes responsible for the biofilm matrix formation, thus, reducing the chances of virulence/resistance-forming genes in *P. aeruginosa* forming.

One of the primary factors determining the virulence of *P. aeruginosa* is pyocyanin, a blue redox-active secondary metabolite that can generate free radicals. Patients with cystic fibrosis frequently experience this substance’s effects, which involve interfering with ion transport and mucus secretion in respiratory epithelial cells [54]. Reactive oxygen species (ROS) produced by pyocyanin significantly impact the development of both acute and chronic respiratory infections and play a crucial role in changing the host immune system [55]. In this study, the observation that the oils significantly reduced the production of pyocyanin in a concentration-dependent manner could be suggestive of their probable anti-pyocyanin effect. At MIC, the fact that both oils reduced pyocyanin in a manner comparable to the reference standards points to their beneficial potential as AQS antimicrobials. This is a significant discovery since pyocyanin synthesis is crucial to the virulence of *P. aeruginosa,* and the observation noted in this study agrees with the report of Pejčić et al. [50], where basil and sage oils significantly decreased pyocyanin production.

The potential of *P. aeruginosa* to colonize various settings through motility influence is another factor supporting its classification as a life-threatening opportunistic pathogen [54]. Motility in *P. aeruginosa* is regulated by QS, where swimming on a soft surface and swarming on a semisolid surface are made possible by flagella and pili IV [32]. A distinct reduction in the diameter zone following the treatment of *P. aeruginosa* cells with the oils revealed their remarkable anti-swarming and swimming motility effect relative to the untreated bacterial cells. The anti-swarming and swimming motility effects demonstrated by the oils were comparable to those displayed by the reference standards. Interestingly, this observation is consistent with the report of Pejčić et al. [50], who found that the swimming and swarming motility were reduced by sage and basil oils. As the swarming and swimming motility of *P. aeruginosa* plays a pivotal role in the emergence, development, and upkeep of the biofilm’s structural framework [56], limiting this mobility may lessen the organism’s virulence ability. This is significant as the oils limited the degree of motility in *P. aeruginosa* in a way that suggested possible interference in the production of virulence factors.

Since the mechanism of action of the oils was unknown, they were profiled using chromatographic techniques to analyze the constituents that might be responsible for the AQS potential observed against *P. aeruginosa* in the in vitro evaluation. The 15 metabolites identified in the oils were essentially fatty acids and vitamins, and the oils could be said to be high linoleic edible oils, as they are rich in polyunsaturated fatty acids (linoleic acids), monounsaturated fatty acids (oleic acid), and others, thus, they are also cholesterol and nutritionally friendly [57].

Emran et al. [58] demonstrated in silico docking as a promising method to support findings from in vitro analysis. Upon identifying the constituents, a molecular docking analysis was performed with all the identified constituents to evaluate which of the compounds are active and bind at the catalytic regions of the Las proteins system of *P. aeruginosa* and the CviR system of *C. violaceum*. Following molecular docking with all the identified constituents from the oils against all the proteins involved in the Las and CviR systems, the compound(s) with the highest negative binding score and interactions were considered to have the greatest affinity for the respective investigated protein [59]. Additionally, the proteins in each case of the Las and CviR systems that were most receptive to the constituents were taken further for analysis. Specifically, the binding affinities from LasR (Las system) and CviR (CviR) proteins were considered as they had the best affinity for the test metabolites. Phylloquinone having the highest negative score against LasR was an indication of its significant affinity for the protein relative to the other test metabolites. This also holds for linoleic acid and oleic acid as the next-ranked metabolites relative to the reference standards and is suggestive of their higher affinity and interactions against LasR. This observation could be due to the higher number of interactions formed between the metabolites and, most especially, phylloquinone with LasR, which was higher than those formed between the reference standards and LasR. This opinion is consistent with the report of Chen et al. [60], where a higher number of interactions and hydrogen bonds of anthocyanins against α–Glucosidase enhanced the affinity of the compound against α–Glucosidase [60]. Furthermore, the top three metabolites interacted with key amino acid residues such as Tyr56, Trp60, Asp73, Ser129 Leu36, Leu40, Tyr47, Val76, and Cys79 in LasR, which is consistent with those reported by Bottomley et al. [61] between LasR’s autoinducer (3–oxo–C12–HSL) and LasR at the catalytic site, with phylloquinone interacting more with these amino acids towards LasR. This is indicative that the top three compounds are binding at the catalytic site of LasR and exhibiting similar interactions and traits as LasR’s autoinducer (3–oxo–C12–HSL). This was similarly observed against CviR, with the top-ranked compounds, including phylloquinone, linoleic acid, myristic acid, and lauric acid, having competitive docking scores and interactions relative to the reference standards, with phylloquinone having the best affinity for the receptor, and suggesting phylloquinone as the best inhibitor of the protein. Measuring the ligand’s RMSD from its reference point in the resulting complex following optimum superimposition is one of the most popular methods for assessing the accuracy of docking geometry [62]. Remarkably, confirmatory superimposition analysis of the docking protocols in this study indicates the same binding position with the native inhibitors of LasR and CviR, hence eliminating the selection of any pseudo-positive binding conformations as the greatest energy-minimized posture. However, due to the limitations of molecular docking, which can only be used as a preliminary investigation of a ligand’s affinity for a protein’s binding pocket, an MD simulation over 100 ns was conducted to gain further insight into LasR and CviR protein’s behavior upon the binding of the top-ranked compounds at the catalytic region. This is important to evaluate the residing time of the metabolites and reference standards at the catalytic region by measuring the binding free energy as well as important conformational information regarding the thermodynamic structural stability, flexibility, and compactness of the complexes taken as post-MD simulation indices [63].

The top three compounds from molecular docking were then further taken to MD simulation. The binding free energy estimates the distinction in energy between a complex and its unbound receptor component, and the higher the negative value, the better the affinity of the ligand toward the enzyme [64]. Phylloquinone, linoleic acid, and oleic acid, when bound with LasR at the catalytic region, had higher negative binding free energy values than the reference standards. This finding suggests the top three compounds as promising and better potential inhibitors of LasR, especially phylloquinone which had the highest negative binding free energy value. Moreover, this observation is suggestive of phylloquinone having a higher residence time at the catalytic region of LasR, thus, resulting in an enhancement of activity towards LasR; it also correlates with the results obtained in the molecular docking studies, where phylloquinone was identified as the most promising metabolite. Similarly, against the CviR of *C. violaceum*, the higher binding free energy values observed with the top four metabolites relative to the cinnamaldehyde point to their advantage as better inhibitors of CviR. While compared to azithromycin, the observation that only phylloquinone and linoleic acid had higher binding free energy values, with phylloquinone having the highest value, demonstrated the benefit of phylloquinone and linoleic acid as an anti-CviR agent, with phylloquinone again having the best inhibitory effect.

The RMSD trajectory was analyzed to evaluate the structural stability of the resulting complexes at the catalytic site of LasR over 100 ns and, thus, the stability of a complex structure is shown by its proximity to the unbound structure, which implies a lower RMSD value [64]. As reported by Ramírez and Caballero [65], a desirable and generally acceptable RMSD number should be less than 3 Å. In this study, the complexes converged at around 15 ns and 45 ns; however, after 60 ns, they were all at equilibration and were relatively stable and compact. The LasR deviated from its native conformation upon the binding of ligand compounds accounting for a maximum fluctuation of less than 2.5 Å average RMSD value, suggesting that all the complexes were within the acceptable RMSD limit of less than 3 Å. This observation points to the thermodynamic structural stability of the top-ranked metabolites towards LasR and further enhances their benefit as AQS agents. Interestingly, the phylloquinone–LasR complex, with the highest negative binding free energy, had the lowest mean RMSD value comparable to the unbound LasR, thus, further indicating the advantage of the compound as a potential LasR inhibitor. Similarly, the observation that only linoleic acid among the top-ranked metabolites against CviR caused increased RMSD relative to unbound CviR suggests the thermodynamic structural stability of top-ranked metabolites with CviR. Moreover, all the RMSD values of the top-ranked metabolites and reference standards were less than 3 Å, with myristic acid having the lowest value.

The RMSF assesses the fluctuation of the amino residues of LasR and CviR protein and can be related to the stability of the intra- and inter-molecular bonds within the complex, and the lower the fluctuation at the catalytic location, the stronger the binding and affinity of the ligand to the protein [66]. The binding of the investigated metabolites to LasR and CviR led to random fluctuations that arose from possible structural conformation changes. For all the metabolites investigated, the lowest fluctuations at the catalytic region of LasR were generally observed between residues 30 and 70. This indicates that metabolite binding at these residues was stable due to fewer fluctuations. This observation is favorable for the top-ranked metabolites and reference standards as inhibitors of LasR and is consistent with the report of Husain et al. [67], where similarly reduced fluctuations in the catalytic region of the protein were observed. This result shows that the three key metabolites from the oils are promising inhibitors of LasR, especially phylloquinone, which is the metabolite that leads to the lowest volatility and flexibility of LasR after binding to the protein, reflecting its increased attractiveness and ability to improve the stability of LasR–amino acid residues. Interestingly, this finding is also coherent with those of the binding free energy. A similar observation was noted for the metabolites and reference standard binding of CviR where, except for linoleic acid and azithromycin, CviR ligand binding caused decreased RMSF. This observation points to the increased attractiveness and ability of the ligands to enhance the stability of CviR amino acid residues. However, in contrast to LasR, where phylloquinone showed the least fluctuation of LasR amino acid residues after its binding, the myristic acid–CviR complex had the least fluctuation. However, phylloquinone–CviR, together with other metabolites and the reference standard binding of CviR, all had an RMSF value that was less than 3 Å, indicating the relatively good fluctuation of the CviR residue after binding of these ligands.

The RoG measures how complexes are thermodynamically compact over time, so the lower the value, the more compact the complex is [68]. In this study, the observation that the binding of the top-ranked metabolites and the reference standards to LasR had a negligible effect on the mean RoG values relative to unbound LasR indicates the relative compactness of the complexes. This means that metabolites’ binding had no thermodynamic perturbation effect on LasR. However, it is worth noting the unfolding effect that azithromycin had on LasR between 70 and 80 ns, suggesting lower compactness and stability within this period. As with LasR, the negligible increasing effect on RoG upon CviR binding by the top four metabolites and reference standard might indicate that the thermodynamic geometry of CviR was not perturbed.

The SASA is a key thermodynamic stability metric that examines protein folding and surface area changes over the simulation, with larger SASA values indicating an increase in protein volume [66]. The physicochemical properties of the amino acid residues that were rearranged or altered determine the degree of variation in the SASA value [69]. Similar to the RoG results, a higher fluctuation of SASA was observed in the azithromycin-LasR and azithromycin–CviR complex plot over the 100 ns simulation period, suggesting that azithromycin has a larger impact on the surface expansion of LasR and CviR protein. However, the comparable SASA value of the unbound proteins (LasR and CviR) and the top-ranked metabolite complexes shows that the LasR and CviR volume either remains the same or decreases over the course of the simulation, thus suggesting no perturbation in protein following the binding of the top-ranked metabolites and cinnamaldehyde during simulation.

In biochemistry, hydrogen bonds are significant interactions as they are crucial for molecular recognition, structural stability, enzyme catalysis, drug partition, and permeability [70,71,72]. Intramolecular hydrogen bonds and distance are important in the stability of a protein structure and, hence, can be assessed to understand the impact of ligand binding on the stability of a protein during simulation [64]. However, the membrane partition and permeability of the medication may be negatively impacted by an excess of hydrogen bond donors or acceptors [73]. In addition to increasing the water desolvation penalty during drug penetration, these polar groups can reduce the attraction for the hydrophobic membrane area [73]. As observed in this study, before and after the top three compounds and reference standards bindings, the stable fluctuation in the pattern of the number of hydrogen bonds produced in LasR and CviR indicates that, following binding to compounds, LasR and CviR thermodynamic entropy was unaffected [19]. In LasR, the decrease in the average number of intramolecular hydrogen bonds created by the top three compounds and controls in complexes with LasR could suggest breakage in some intramolecular hydrogen bonds due to ligand binding. This observation is unlike what was reported by Aribisala and Sabiu [33] and those observed in this study with CviR, where top-ranked metabolites and reference standards binding of CviR caused increased intramolecular hydrogen bonds. The increased intramolecular hydrogen bonds of CviR could be due to the addition of intermolecular hydrogen bonds contributed by ligand binding. However, the high number of intramolecular hydrogen bonds noted with phylloquinone–LasR and phylloquinone–CviR protein corroborates the stability observed with the complex, which implies that there was a high degree of thermodynamic compatibility during the 100 ns simulation duration, which may have caused the higher binding free energy observed with the compound against LasR and CviR.

The top-ranked compounds and the reference standards exhibited different bond lengths and numbers of interactions in the active site of LasR and CviR, which were found to have an impact on the free energy of binding observed in this study. The highest number of interactions and hydrogen bonding contacts observed with the phylloquinone–LasR and the phylloquinone–CviR complexes after 100 ns is consistent with the binding free energy observed with the metabolites against the respective target. This observation indicated that a stronger and more stable ligand–protein complex results from more interactions and hydrogen bonding contacts, suggesting greater inhibition in the phylloquinone–LasR and phylloquinone–CviR complexes observed in this study. This conclusion agrees with the observation of Forli et al. [74], who showed that the higher the number of interactions, including hydrogen bonding, the higher the inhibitory effect of the inhibitor. Similar observations were noted with the other top-ranked metabolites compared to the reference standards against LasR and CviR, where top-ranked metabolites with higher binding free energy showed a higher number of interactions and hydrogen bonding contacts. In addition to the highest number of hydrogen bonding contacts, phylloquinone–LasR and phylloquinone–CviR complexes had the highest number of important interactions, such as the stacking interaction (stacked), which is one of the most powerful driving forces behind the biological complexation process, and protein folding. Taken together, all of these observations may have contributed to the observed higher affinity and improved stability between phylloquinone and the investigated targets. Since analyzing the number and type of interactions in just one simulation time frame could lead to a false positive conclusion, snapshots at the different time frames of the simulation were taken, and the observation that the consistent number of interactions and some conserved residues exist at 30 ns, 60 ns, and 100 ns during the simulation point to the potential enhanced inhibitory effect on the proteins by the top-ranked metabolites and reference standards during the 100 ns simulation period.

To avoid or reduce the tendency of high failure rate during the preclinical and clinical phases of drug development, evaluation of the pharmacokinetics, drug likeliness, synthetic feasibility, and toxicity characteristics of potential therapeutic agents have been identified as a critical step and a prerequisite, and this was undertaken for the top-ranked metabolites in this study. The rule of 5 (RO5), also known as Lipinski’s rule, provides a useful framework to determine whether a tested molecule will be orally accessible and bioavailable [75]. It also affirms that molecules exhibit strong absorption or permeation if they have an octanol/water partition coefficient (log P) < 5, molecular weight (MW) < 500 g/mol, number of hydrogen bond donors (n OH, NH) < 5, and number of hydrogen bond acceptors (n O, N) ≤ 10 [76]. Interestingly, the top-ranked metabolites fulfilling Lipinski’s rule showed their ability to be orally administrable to reach target sites and exert their pharmacological effects, while azithromycin failing the rule suggests its relatively lower tendency to be orally administered and, thus, pinpoints the probable advantage of the top-ranked compounds over azithromycin as drug candidates. However, this does not imply that azithromycin is not a good drug but suggests that it can nonetheless be modified to improve its administration via the oral route, which is mostly preferred [77]. On the other hand, while the low GIT absorption rate of azithromycin and phylloquinone indicates that they are less likely to be absorbed through the GIT, the high GIT absorption rate of linoleic acid and oleic acid indicates their advantage over azithromycin and phylloquinone. Azithromycin, with the lowest bioavailability score, indicates that it is not preferred as an oral drug over cinnamaldehyde, and the top-ranked compounds with higher bioavailability scores mean a high rate of absorption of a drug and the concentration of unchanged drug that reaches the site of action to exert its pharmacological effect. This observation agrees with the findings of Shode et al. [78] regarding anti-COVID-19 drug candidates with relatively higher bioavailability scores than the reference standards used. Additionally, phylloquinone and the other top-ranked compounds had bioavailability ratings comparable to cinnamaldehyde, which suggests that they can be used orally and will be bioavailable since cinnamaldehyde is an oral drug. Interestingly, the top-ranked compounds, except phylloquinone, were also water-soluble, suggesting their advantage of being easily transported through the bloodstream compared to azithromycin, which is poorly soluble in water.

## 5. Conclusions

The results of this investigation demonstrated the effectiveness of oils in modulating quorum sensing of *P. aeruginosa*, which subsequently prevented biofilm formation and other virulence factors. Interestingly, the anti-QS potential observed with the oils competed favorably well with conventional drugs used as standard in vitro. Findings from the in silico study revealed linoleic acid, oleic acid, and especially phylloquinone to be mostly responsible for the high anti-QS activity observed with the oils as they were able to bind at the active site of LasR and relatively stable over 100 ns simulation period. Noteworthily, bioprospecting of the oil constituents against the CviR QS system of the biomonitor strain, *C. violaceum,* revealed, in particular, the ability of phylloquinone and linoleic acid to bind and inactivate the protein, suggesting the broad spectrum AQS capacity of the lead metabolites. Fascinatingly, the top-ranked compounds also passed Lipinski’s rule of five, indicating their drug-likeness properties. Consequently, the top-ranked metabolites of the oils, and most particularly phylloquinone, may function as a potential LasR modulator and constitute a novel therapeutic drug candidate for infections caused by *P. aeruginosa*. As a result, phylloquinone could be studied further as a QS modulator and perhaps find utility in the development of new therapeutics.

## Figures and Tables

**Table 1:** Minimum inhibitory concentrations of the treatments on *P. aeruginosa*.

Treatment	MIC (mg/mL)
Azithromycin	0.25
Cinnamaldehyde	3.75
2% DMSO	0.00
AGSUN 5102 CLP	91.80
AGSUN 5106 CLP	91.80

**Table 2:** Qualitative AQS activity of AGSUN 5102 CLP and AGSUN 5106 CLP essential oils against *Chromobacterium violaceum* 12472 at sub-MIC concentrations (mg/mL).

Zone Diameters (mm) and Related Susceptibility Traits		
Concentration (mg/mL)	AGSUN 5102 CLP	AGSUN 5106 CLP
91.80	2.33	1.00
45.90	0.00	0.00
22.95	0.00	0.00
11.48	0.00	0.00
	Controls	
Concentration (mg/mL)	Azithromycin	
0.25	13.33	
0.13	0.00	
0.06	0.00	
0.03	0.00	
Concentration (mg/mL)	Cinnamaldehyde	
3.75	5.33	
1.88	0.00	
0.94	0.00	
0.47	0.00	
2% DMSO	0.00	

Intermediate = 11–14 mm and Resistant ≤ 10 mm.

**Table 3:** Inhibitory effect of essential oils on QS-mediated virulence factor swarming motility of *P. aeruginosa* ATCC 27853 at sub-MIC concentrations (mg/mL).

Zone Diameters (mm)		
Concentration (mg/mL)	AGSUN 5102 CLP	AGSUN 5106 CLP
91.80	2.0	3.0
45.90	2.5	3.5
22.95	3.0	4.3
11.48	4.4	4.8
	Controls	
Concentration (mg/mL)	Azithromycin	
0.25	2.0	
0.13	2.3	
0.06	2.8	
0.03	3.5	
Concentration (mg/mL)	Cinnamaldehyde	
3.75	2.5	
1.88	3.0	
0.94	3.8	
0.47	4.5	
2% DMSO	5.0	
Untreated cells	5.0	

**Table 4:** Inhibitory effect of essential oils on QS-mediated virulence factor swimming motility of *P. aeruginosa* ATCC 27853 at sub-MIC concentrations (mg/mL).

Zone diameters (mm)		
Concentration (mg/mL)	AGSUN 5102 CLP	AGSUN 5106 CLP
91.80	21.0	23.0
45.90	26.0	28.0
22.95	29.0	30.3
11.48	31.0	33.0
	Controls	
Concentration (mg/mL)	Azithromycin	
0.25	17.0	
0.13	22.0	
0.06	26.3	
0.03	30.0	
Concentration (mg/mL)	Cinnamaldehyde	
3.75	22.0	
1.88	26.5	
0.94	29.0	
0.47	32.0	
2% DMSO	34.0	
Untreated cells	34.0	

**Table 5:** Results of chromatographic analysis of AGSUN 5102 CLP and AGSUN 5106 CLP oils.

Chromatographic Technique Used	Carbon Length and Degree of Saturation	Metabolites	Molecular Weight (g/mol)	Molecular Formula	Concentration (µg/mL)	
					AGSUN 5102 CLP	AGSUN 5106 CLP
GCMS	C6	Caproic acid	116.16	C_6_H_12_O_2_	31.63	27.01
GCMS	C8	Caprylic acid	144.21	C_8_H_16_O_2_	193.32	133.08
GCMS	C10	Capric acid	173.26	C_10_H_12_O_2_	26.77	5.40
GCMS	C12	Lauric acid	200.32	C_12_H_24_O_2_	16.55	4.61
GCMS	C14	Myristic acid	228.37	C_14_H_28_O_2_	43.37	30.92
GCMS	C15	Pentadecylic acid	242.40	C_15_H_30_O_2_	14.01	10.67
GCMS	C16	Palmitic acid	256.42	C_16_H_32_O_2_	3690.16	2590.31
GCMS	C18	Stearic acid	284.50	C_18_H_36_O_2_	4675.40	3228.19
GCMS	C18:1 cis	Oleic acid	282.47	C_18_H_34_O_2_	13,102.20	12,283.50
GCMS	C18:2 cis	Linoleic acid	280.45	C_18_H_32_O_2_	32,181.45	17,833.84
GCMS	C20	Arachidic acid	312.50	C_20_H_40_O_2_	250.45	186.03
GCMS	C22	Behenic acid	340.60	C_22_H_44_O_2_	546.09	465.99
GCMS	C23	Tricosylic acid	354.60	C_23_H_46_O_2_	21.03	18.63
GCMS	C24	Lignoceric acid	368.60	C_24_H_48_O_2_	126.24	105.36
HPLC	C31	Phylloquinone	450.70	C_31_H_46_O_2_	0.059	0.054

**Table 6:** Binding affinities and bonds formed between the top three compounds and LasR protein.

Compounds	Binding Score (kcal/mol)	Total Bonds	Hydrogen Bonds	Other Significant Bonds
Azithromycin	−6.3	16	4 (Lys16, Ser20, Glu48, Gly54)	-
Cinnamaldehyde	−7.4	13	-	4 (Trp88, Phe101, Ala105, Leu110)
Phylloquinone	−9.4	29	3 (Tyr56, Trp60, Ser129)	12 (Leu40, Tyr47, Ala50, Tyr64, Ala70, Asp73, Val76, Cys79, Leu105, Leu110, Leu125, Ala127)
Linoleic acid	−8.5	24	-	13 (Leu36, Leu40, Tyr47, Ala50, Ile52, Tyr56, Trp60 Ala70, Val76, Phe101, Leu110, Leu1250
Oleic acid	−8.2	25	-	13 (Leu36, Leu40, Tyr47, Ala50, Ile52, Tyr56, Trp60, Ala70, Val76, Phe101, Leu110, Leu125)

**Table 7:** Energy components (kcal/mol) of the top three compounds against LasR of *P. aeruginosa*.

Target	Ligands	ΔE_vdW_	ΔE_elec_	ΔG_gas_	ΔG_solv_	ΔG_bind_
*P. auruginosa* + LasR	Cinnamaldehyde	−23.55 ± 1.75	−15.11 ± 2.54	−38.67 ± 2.52	21.72 ± 1.78	−16.95 ± 1.75
	Azithromycin	−24.92 ± 13.09	−251.03 ± 53.07	−289.22 ± 63.07	257.13 ± 54.76	−32.08 ± 10.54
	Phylloquinone	−70.70 ± 4.59	−17.35 ± 2.80	−88.06 ± 5.33	21.64 ± 1.62	−66.42 ± 4.63
	Linoleic acid	−51.12 ± 3.05	−25.18 ± 4.43	−76.31 ± 4.56	23.16 ± 2.46	−53.14 ± 3.53
	Oleic acid	−55.03 ± 3.19	−15.08 ± 7.15	−70.12 ± 8.19	18.10 ± 6.47	−52.02 ± 3.91

ΔE_vdW_ = van der Waals energy; ΔG_bind_ = total binding free energy; ΔE_gas_ = gas phase free energy; ΔE_elec_ = electrostatic energy; ΔG_solv_ = solvation free energy.

**Table 8:** Average RMSD, ROG, RMSF, SASA, and intramolecular hydrogen bond number of the top-three compounds against LasR of *P. aeruginosa* following a 100-ns simulation.

Target	Ligands	RMSD (Å)	RMSF(Å)	ROG (Å)	SASA (Å)	Intramolecular H–Bond
*P. aeruginosa* LasR +	Apo LasR	1.48 ± 0.11	1.04 ± 0.41	15.32 ± 0.07	8546.51 ± 204.30	83.20 ± 6.20
	Cinnamaldehyde	1.41 ± 0.19	1.18 ± 0.59	15.36 ± 0.07	8680.70 ± 224.56	81.44 ± 6.40
	Azithromycin	1.75 ± 0.24	1.29 ± 1.38	15.58 ± 0.32	9090.31 ± 325.34	79.33 ± 6.20
	Phylloquinone	1.51 ± 0.18	1.11 ± 0.57	15.57 ± 0.08	8623.64 ± 242.77	81.24 ± 6.60
	Linoleic acid	1.87 ± 0.25	1.25 ± 0.66	15.47 ± 0.09	8629.81 ± 239.72	79.44 ± 6.50
	Oleic acid	1.96 ± 0.33	1.55 ± 0.64	15.36 ± 0.07	8729.91 ± 269.80	78.05 ± 6.40

**Table 9:** Cheminformatics properties of the top-ranked compounds against LasR.

Property	Azithromycin	Cinnamaldehyde	Phylloquinone	Linoleic acid	Oleic Acid
Molecular weight (g/mol)	748.996	132.160	450.700	280.447	282.470
Bioavailability score	0.17	0.55	0.55	0.85	0.85
Water solubility	Poorly soluble	Soluble	Poorly soluble	Moderately soluble	Moderately soluble
Lipophilicity	4.76	1.65	6.01	4.17	4.27
GIT absorption	Low	High	Low	High	High
BBB permeability	No	Yes	No	Yes	No
Hydrogen bond acceptors	14	1	2	2	2
Hydrogen bond donors	5	0	0	1	1
Lipinski’s rule	No (3 violations: MW > 500 g/mol, N or O > 10, MLOGP > 4.15)	Yes	Yes (1 violation: MLOGP > 4.15)	Yes (1 violation: MLOGP > 4.15)	Yes (1 violation: MLOGP > 4.15)

## Data Availability

The data presented in this study are available in the article or its Supplementary file.

## Supplementary Materials

The following supporting information can be downloaded at: https://www.mdpi.com/article/10.3390/antibiotics12030504/s1, Figure S1: Qualitative anti-quorum sensing activity of (a) 2% DMSO, (b) azithromycin, (c) cinnamaldehyde, (d) AGSUN 5102 CLP, and (e) AGSUN 5106 CLP against a biomonitor strain of *C. violaceum* at different concentrations; Table S1. Chromatography results of the identified metabolites of AGSUN 5102 CLP and AGSUN 5106 CLP essential oils; Figure S2. Inhibitory effect of the controls (treated and untreated), (a) untreated cells, (b) azithromycin, (c) cinnamaldehyde on QS-mediated virulence factor swarming motility of *P. aeruginosa* ATCC 27853 at MIC [0.25 mg/mL (azithromycin) and 3.75 mg/mL (cinnamaldehyde)], (d) AGSUN 5102 CLP, and (e) AGSUN 5106 CLP on QS-mediated virulence factor swarming motility of *P. aeruginosa* ATCC 27853 at 91.80 mg/mL; Table S2. Binding affinities of the metabolites against all the proteins involved in the Las QS system of *P. aeruginosa*; Figure S3: Inhibitory effect of the reference standards (treated and untreated), (a) untreated cells, (b) azithromycin at 0.25 mg/mL, (c) cinnamaldehyde at 3.75 mg/mL, (d) AGSUN 5102 CLP at 91.80 mg/mL, and (e) AGSUN 5106 CLP at 91.80 mg/mL on QS-mediated virulence factor swarming motility of *P. aeruginosa* ATCC 27853; Table S3. Binding affinities of the metabolites against LasR; Figure S4. (a) Root mean square deviation (RMSD), (b) root mean square fluctuations (RMSF), (c) radius of gyration (RoG), (d) solvent accessible surface area (SASA), and (e) number of intramolecular hydrogen bonds with time plots of comparison between CviR and top four compounds, azithromycin, and cinnamaldehyde determined over 100 ns molecular dynamics simulations; Table S4. Interactions formed in the binding of the reference standards and metabolites from the essential oils and LasR; Table S5. Binding affinities of the metabolites against the proteins involved in the CviR quorum sensing of *C. violaceum;* Table S6. Binding affinities of the metabolites and CviR; Table S7. Interactions formed in the binding of metabolites and CviR; Table S8. Energy components (kcal/mol) after 100 ns MDS; Table S9. RMSD, ROG, RMSF, SASA, H–Bonds after 100 ns MDS; Table S10: Plots of interactions of top-ranked metabolites and reference standard towards LasR and CviR at different time intervals during the 100 ns MD simulation.
