# Supplementary material for: Cheminformatics Bioprospection of Sunflower Seeds’ Oils against Quorum Sensing System of Pseudomonas aeruginosa
Source: Antibiotics (Basel). 2023 Mar 2;12(3):504. doi: 10.3390/antibiotics12030504 (PMC10044302; doi:10.3390/antibiotics12030504)
Supplement: Supplementary file 1 [file antibiotics-12-00504-s001.zip › antibiotics-2210079-supplementary.pdf]

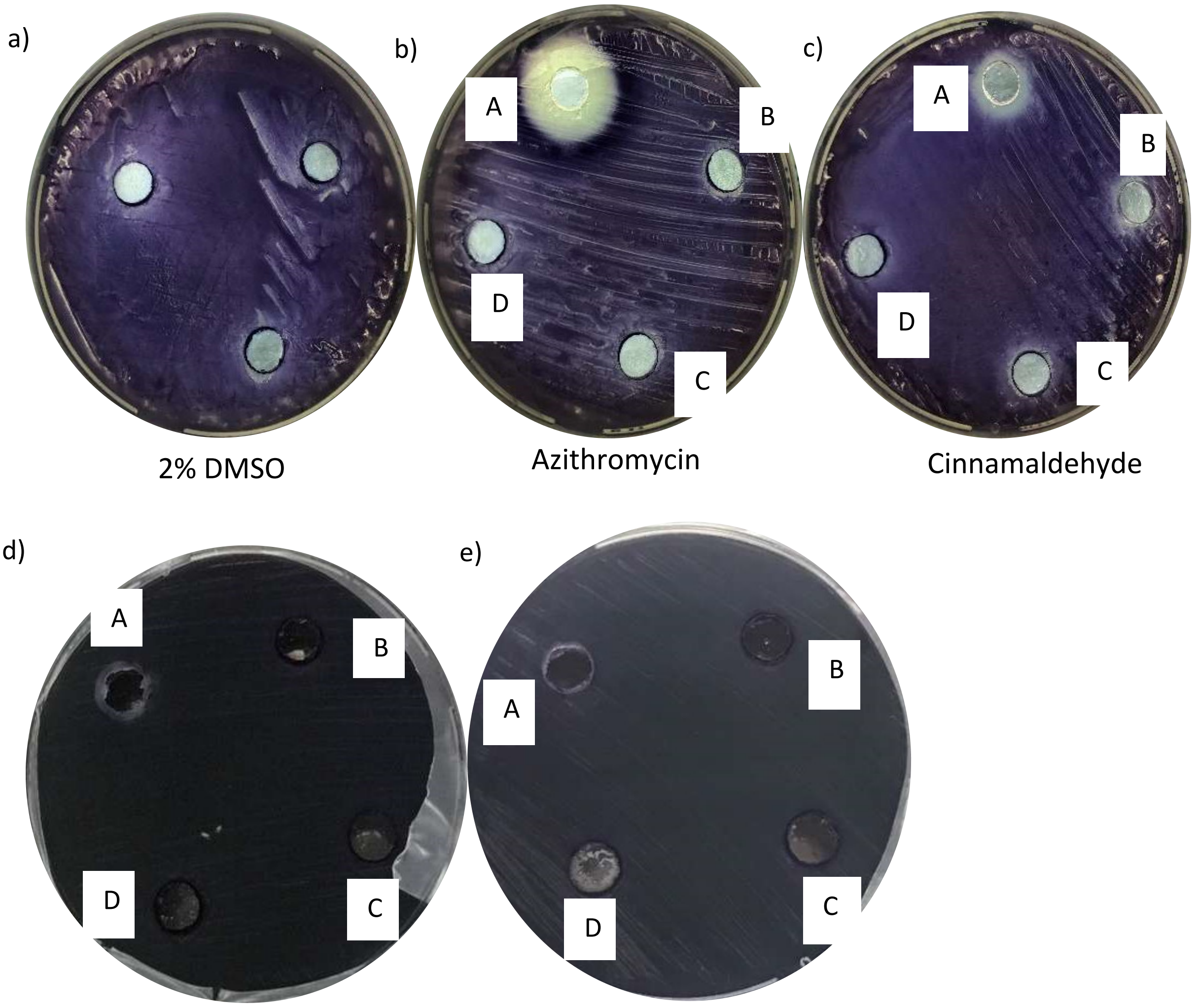

Keys: [illegible] MIC; **D**: 1/8 MIC

Figure S1. Qualitative anti-quorum sensing activity of (a) 2% DMSO, (b) azithromycin, (c) cinnamaldehyde, (d) AGSUN 5102 CLP and (e) AGSUN 5106 CLP against a biomonitor strain of *C. violaceum* at different concentrations.

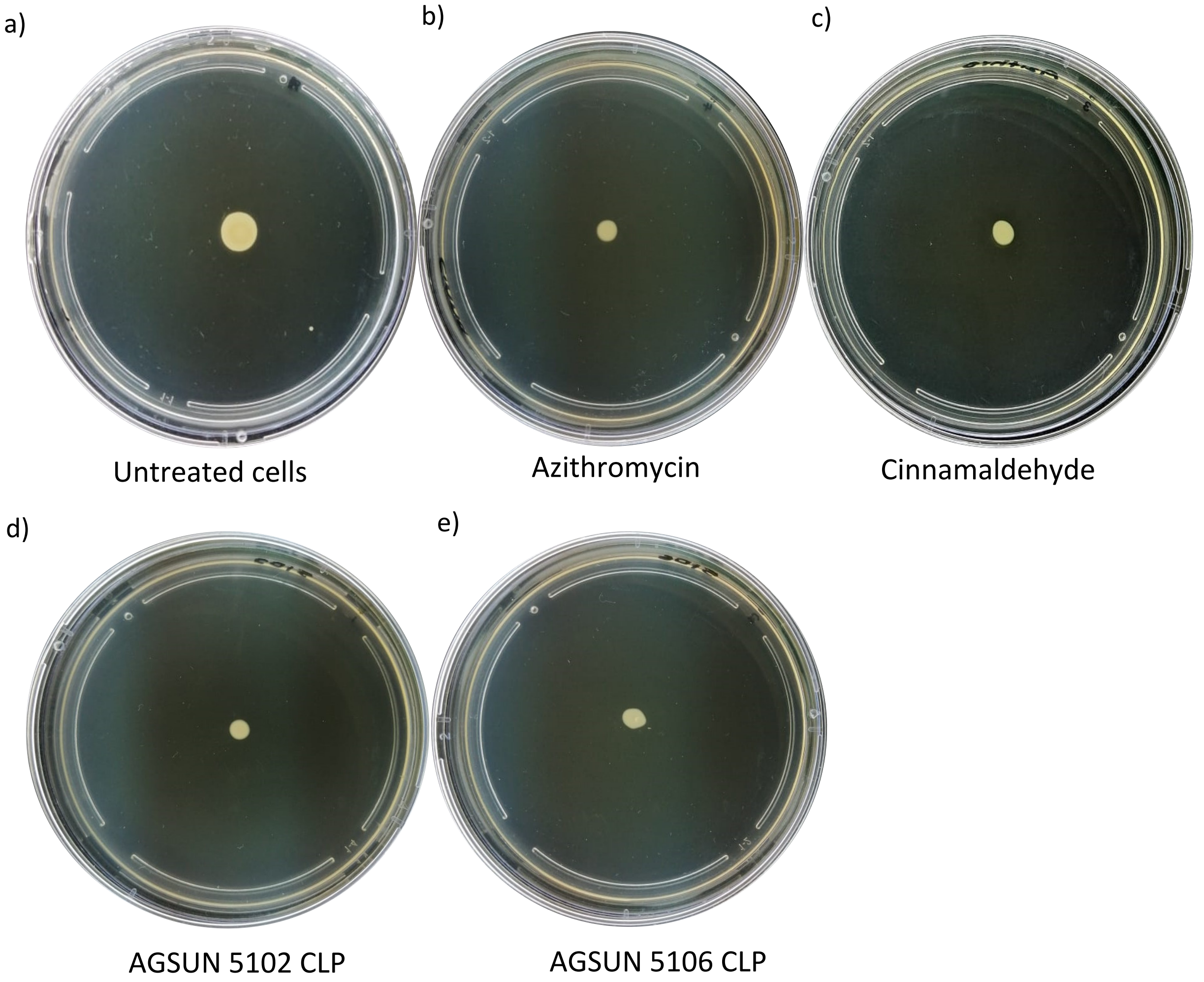

Figure S2. Inhibitory effect of the controls (treated and untreated), (a) untreated cells, (b) azithromycin (c) cinnamaldehyde on QS–mediated virulence factor swarming motility of *P. aeruginosa* ATCC 27853 at MIC [0.25 mg/mL (azithromycin) and 3.75 mg/mL (cinnamaldehyde)], (d) AGSUN 5102 CLP and (e) AGSUN 5106 CLP on QS–mediated virulence factor swarming motility of *P. aeruginosa* ATCC 27853 at 91.80 mg/mL.

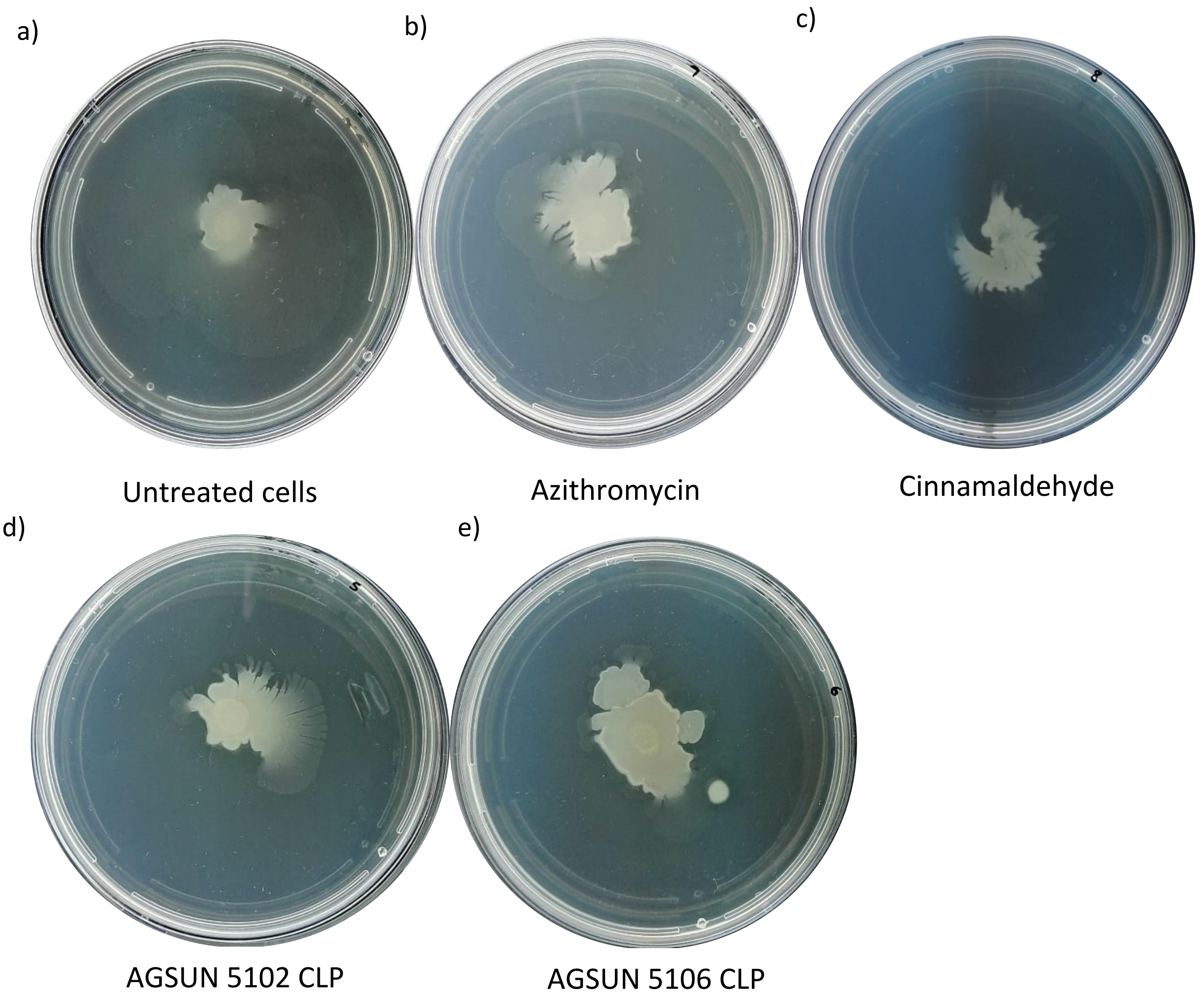

Figure S3. Inhibitory effect of the reference standards (treated and untreated), (a) untreated cells, (b) azithromycin at 0.25 mg/mL, (c) cinnamaldehyde at 3.75 mg/mL, (d) AGSUN 5102 CLP at 91.80 mg/mL and (e) AGSUN 5106 CLP at 91.80 mg/mL on QS–mediated virulence factor swarming motility of *P. aeruginosa* ATCC 27853.

Table S1. Chromatography results of the identified metabolites of AGSUN 5102 CLP and AGSUN 5106 CLP essential oils

**Carbon length & degree of saturation**	**Metabolites**	**Retention time**	**M/Z ratio**		
				**AGSUN 5102 CLP**	**AGSUN 5106 CLP**
C6	Caproic acid	10.45	28.50	31.63	27.01
C8	Caprylic acid	13.96	32.00	193.32	133.08
C10	Capric acid	18.12	35.00	26.77	5.40
C12	Lauric acid	23.89	37.10	16.55	4.61
C14	Myristic acid	28.48	38.20	43.37	30.92
C15	Pentadecyclic acid	31.30	23.00	14.01	10.67
C16	Palmitic acid	33.88	52.00	3690.16	2590.31
C18	Stearic acid	38.00	38.50	4675.40	3228.19
C18:1 cis	Oleic acid	39.00	40.05	13102.20	12283.50
C18:2 cis	Linoleic acid	40.95	25.00	32181.45	17833.84
C20	Arachidic acid	42.00	36.00	250.45	186.03
C22	Behenic acid	46.00	34.00	546.09	465.99
C23	Tricosylic acid	47.90	20.02	21.03	18.63
C24	Lignoceric acid	48.90	34.00	126.24	105.36

Table S2. Binding affinities of the metabolites against all the proteins involved in the Las QS system of *P. aeruginosa*

Carbon length	Metabolites	Binding affinities (kcal/mol)					
		LasR	LasA	LasB	LasI	ToxA	AprA
C9	Cinnamaldehyde	-7.4	-4.8	-5.2	-3.5	-6.1	-5.2
C38	Azithromycin	-6.3	-6.3	-6.9	-2.9	-5.3	-6.9
C6	Caproic acid	-5.5	-4.6	-4.5	-3.9	-5.0	-4.5
C8	Caprylic acid	-5.9	-5.0	-4.6	-3.6	-5.1	-5.0
C10	Capric acid	-6.3	-5.3	-4.9	-3.3	-5.3	-4.9
C12	Lauric acid	-7.0	-5.3	-5.0	-3.9	-5.7	-5.0
C14	Myristic acid	-7.1	-5.3	-4.9	-3.9	-5.3	-5.4
C15	Pentadecyclic acid	-7.5	-5.1	-4.7	-4.0	-5.6	-5.4
C16	Palmitic acid	-7.7	-5.1	-5.3	-4.1	-5.6	-5.2
C18	Stearic acid	-7.9	-5.2	-5.3	-4.1	-5.6	-4.8
C18:1 cis	Oleic acid	-8.2	-5.0	-4.8	-4.1	-5.6	-5.6
C18:2 cis	Linoleic acid	-8.5	-5.4	-5.2	-4.4	-5.9	-5.7
C20	Arachidic acid	-7.9	-5.0	-5.6	-3.8	-5.6	-5.0
C22	Behenic acid	-7.9	-5.0	-4.8	-4.0	-6.1	-5.0
C23	Tricosylic acid	-7.9	-5.1	-4.9	-3.9	-5.9	-5.2
C24	Lignoceric acid	-7.8	-5.0	-4.7	-3.8	-5.8	-5.9
C31	Phylloquinone	-9.4	-6.3	-6.7	-4.2	-8.0	-7.2

Table S3. Binding affinities of the metabolites against LasR

Carbon length & degree of saturation	Metabolites	Score (kcal/mol)	pKi (μM)
C9	Cinnamaldehyde	-7.4	5.44
C38	Azithromycin	-6.3	4.63
C31	Phylloquinone	-9.4	6.92
C18:2 cis	Linoleic acid	-8.5	6.25
C18:1 cis	Oleic acid	-8.2	6.03
C18	Stearic acid	-7.9	5.81
C20	Arachidic acid	-7.9	5.81
C22	Behenic acid	-7.9	5.81
C23	Tricosylic acid	-7.9	5.81
C24	Lignoceric acid	-7.8	5.74
C16	Palmitic acid	-7.7	5.66
C15	Pentadecyclic acid	-7.5	5.52
C14	Myristic acid	-7.1	5.22
C12	Lauric acid	-7.0	5.15
C10	Camphene	-6.7	4.93
C10	Capric acid	-6.3	4.63
C8	Caprylic acid	-5.9	4.34
C6	Caproic acid	-5.5	4.05

Table S4. Interactions formed in the binding of the reference standards and metabolites from the essentials oils and LasR

**Metabolites**	**Interactions**					**Average bond length**
	Van der waals	Conventional/Donor hydrogen bonds	Pi–Anion/Pi–Pi–Sigma	Alkyl/Pi–Alkyl Pi–Pi stacked/ Pi–Pi T–shaped	Unfavourable Acceptor–Acceptor	
Cinnamaldehyde	Tyr56<br>Tyr64<br>Tyr93<br>Thr75<br>Trp60<br>Ser56<br>Leu36<br>Val76<br>Asp73	–		Ala105<br>Leu110<br>Trp88<br>Phe101		6.43
Azithromycin	Leu17<br>Val53<br>Ala50<br>Ala58<br>Asn55<br>Asn49<br>Arg61<br>Arg66<br>Asp65<br>Ile52<br>Glu62<br>Try56	Lys16<br>Ser20<br>Glu48<br>Gly54				5.63
Phylloquinone	Tyr93<br>Phe101<br>Glu48<br>Asp65<br>Ile52<br>Gly38<br>Gly126<br>Arg61<br>Leu36<br>Leu39<br>Thr75<br>Thr80<br>Thr115<br>Trp88	Tyr56<br>Trp60<br>Ser129	Asp73<br>Tyr64	Leu40<br>Leu110<br>Leu125<br>Ala50<br>Ala70<br>Ala105<br>Ala127<br>Cys79<br>Tyr47<br>Val76	–	5.71
Linoleic acid	Leu39<br>Gly36	–	–	Leu36	–	5.99

	Gly126<br>Ala105<br>Ala127<br>Trp88<br>Tyr93<br>Thr75<br>Asp73<br>Ser129<br>Arg61			Leu40 Tyr47<br>Ala50<br>Ile52 Tyr56<br>Trp60<br>Ala70<br>Val76 Phe101<br>Leu110<br>Leu125		
Oleic acid	Leu40<br>Gly38<br>Gly126<br>Trp88<br>Ala105<br>Phe101<br>Phe102<br>Tyr93<br>Thr75<br>Asp73<br>Ser129<br>Arg61	–	–	Leu36 Tyr47<br>Ala50<br>Ile52<br>Tyr56<br>Trp60<br>Tyr64 Ala70<br>Val76 Cys79<br>Leu110<br>Leu125<br>Ala127	–	5.60
Stearic acid	Gly36<br>Gly126<br>Ser129<br>Asp73<br>Tyr56<br>Tyr93<br>Thr75<br>Arg61<br>Trp60<br>Trp88<br>Phe101<br>Phe102<br>Ala105<br>Leu110	–	–	Val76<br>Leu36<br>Leu40<br>Leu125<br>Ala50<br>Ala70<br>Ala127<br>Tyr47<br>Tyr64<br>Ile52	–	5.31

Arachidic acid	Gly38<br>Gly126<br>Leu39<br>Leu110<br>Ile52<br>Ile92<br>Ser129<br>Trp60<br>Trp88<br>Asp73<br>Thr75<br>Ala105<br>Phe101	Tyr93	–	Cys79<br>Ala50<br>Ala70<br>Ala127<br>Leu36<br>Leu40<br>Leu125<br>Tyr47<br>Tyr56<br>Tyr64<br>Val76	–	5.40
Behenic acid	Gly126<br>Ile52<br>Trp60<br>Trp88<br>Ala105<br>Tyr93<br>Phe101<br>Thr75<br>Asp73<br>Ser129<br>Gly38<br>Arg61	Leu110	–	Tyr47<br>Tyr56<br>Tyr64<br>Leu36<br>Leu40<br>Leu125<br>Ala50<br>Ala70<br>Ala127<br>Cys79<br>Val76	–	5.59
Tricosylic acid	Gly38<br>Gly126	–	–	Leu36<br>Leu40	–	5.68

	Arg61<br>Ile52<br>Asp65<br>Ser129<br>Thr75<br>Asp73<br>Trp88<br>Ala105<br>Tyr93<br>Phe102			Leu110<br>Leu125<br>Cys79<br>Tyr47<br>Tyr56<br>Tyr64<br>Ala50<br>Ala70<br>Ala127<br>Val76<br>Trp60<br>Phe101		
Lignoceric acid	Leu39<br>Leu110<br>Gly38<br>Gly126<br>Thr80<br>Arg61<br>Ser129<br>Ala105<br>Thr75<br>Thr115<br>Asp73<br>Trp88<br>Pro74<br>Ile92<br>Phe101	Tyr93	–	Cys79<br>Leu36<br>Leu40<br>Leu125<br>Val76<br>Tyr47<br>Tyr56<br>Tyr64<br>Ala50<br>Ala70<br>Ala127<br>Ile52<br>Trp60	–	5.43
Palmitic acid	Gly38<br>Gly126	–	–	Leu36<br>Leu40	–	5.82

	Leu39<br>Leu110<br>Tyr47<br>Arg61<br>Ser129<br>Asp73<br>Phe101<br>Phe102<br>Trp60<br>Trp88<br>Thr75<br>Ala105<br>Tyr93			Ala50<br>Ala70<br>Ala127<br>Val76<br>Tyr56<br>Tyr64<br>Ile52		
Pentadecyclic acid	Gly38<br>Gly126<br>Arg61<br>Ser129<br>Asp73<br>Trp60<br>Phe101<br>Thr75<br>Val111<br>Ala105<br>Tyr93	Leu110	–	Leu36<br>Leu40<br>Ala50<br>Ala70<br>Tyr47<br>Tyr56<br>Tyr64<br>Val76<br>Ile52<br>Trp88	–	5.41
Myristic acid	Leu40<br>Leu110<br>Gly38<br>Gly126<br>Arg61<br>Thr115	Ser129	–	Leu36<br>Leu125<br>Tyr47<br>Cys79<br>Ala50<br>Ala70	Thr75	5.22

	Asp73<br>Trp88<br>Tyr56			Ala127<br>Ile52<br>Tyr64<br>Val76		
Lauric acid	Gly38<br>Arg61<br>Ser129<br>Phe101<br>Phe102<br>Asp73<br>Thr75<br>Trp88<br>Ala105<br>Tyr93	–	–	Tyr47<br>Ala50<br>Ala70<br>Ile52<br>Leu36<br>Leu110<br>Tyr56<br>Tyr64<br>Trp60	–	5.74
Capric acid	Gly38<br>Gly126<br>Arg61<br>Thr75<br>Thr115<br>Asp73	Tyr56<br>Ser129	–	Leu36<br>Leu40<br>Leu125<br>Tyr47<br>Tyr64<br>Val76<br>Ile52<br>Ala50<br>Ala70	–	5.16
Caprylic acid	Tyr93<br>Leu36<br>Thr75<br>Thr115<br>Ala127<br>Val76<br>Asp73	Ser129	Trp88	Phe101<br>Ala105<br>Leu110<br>Trp60<br>Tyr56<br>Tyr64	–	5.40

Caproic acid	Leu36<br>Tyr56<br>Tyr64<br>Tyr93<br>Trp60<br>Thr75<br>Asp73	Thr115<br>Ser129	Trp88	Phe101<br>Ala105<br>Leu110	–	4.67

Table S5. Binding affinities of the metabolites against the proteins involved in the CviR quorum sensing of *C. violaceum*

Carbon length & degree of saturation	Metabolites	Binding affinities (kcal/mol)	
		CviR	VioA
C9	Cinnamaldehyde	-7.1	-5.9
C38	Azithromycin	-5.3	-7.5
C6	Caproic acid	-5.4	-4.4
C8	Caprylic acid	-5.6	-4.9
C10	Capric acid	-6.2	-5.2
C12	Lauric acid	-6.6	-5.8
C14	Myristic acid	-6.6	-6.1
C15	Pentadecyclic acid	-6.5	-5.9
C16	Palmitic acid	-6.5	-6.0
C18	Stearic acid	-6.3	-5.3
C18:1 cis	Oleic acid	-6.5	-6.4
C18:2 cis	Linoleic acid	-6.7	-6.7
C20	Arachidic acid	-6.4	-6.4
C22	Behenic acid	-6.5	-6.3
C23	Tricosylic acid	-6.5	-6.4
C24	Lignoceric acid	-6.2	-6.6
C31	Phylloquinone	-8.6	-9.0

Table S6. Binding affinities of the metabolites and CviR

**Carbon length & degree of saturation**	**Metabolites**	**Score (kcal/mol)**	**pKi (μM)**
C9	Cinnamaldehyde	-7.1	5.22
C38	Azithromycin	-5.3	3.90
C31	Phylloquinone	-8.6	6.33
C18:2 cis	Linoleic acid	-6.7	4.93
C14	Myristic acid	-6.6	4.86
C12	Lauric acid	-6.6	4.86
C15	Pentadecyclic acid	-6.5	4.78
C16	Palmitic acid	-6.5	4.78
C18:1 cis	Oleic acid	-6.5	4.78
C22	Behenic acid	-6.5	4.78
C23	Tricosylic acid	-6.5	4.78
C20	Arachidic acid	-6.4	4.71
C18	Stearic acid	-6.3	4.63
C24	Lignoceric acid	-6.2	4.56
C10	Capric acid	-6.2	4.56
C10	Camphene	-5.9	4.34
C8	Caprylic acid	-5.6	4.12
C6	Caproic acid	-5.4	3.97

Table S7. Interactions formed in the binding of metabolites and CviR

Metabolites	Interactions					Average Bond lengths
	Van der waals	Hydrogen bonds	Alkyl/Pi–Alkyl /Pi–Pi stacked	Pi–sigma/Pi anion	Unfavourable Donor–Donor	
Cinnamaldehyde	Phe115<br>Phe126<br>Trp84<br>Tyr80<br>Tyr88<br>Leu57<br>Leu100<br>Ile153	Ser155	Ile93<br>Ala130<br>Met135<br>Trp111	Asp97		5.68
Azithromycin	Gly134<br>Gly136<br>Gly158<br>Gln132<br>Thr131<br>Asn116<br>Ile127<br>Phe115<br>Glu112<br>Ser137	Arg159<br>Ala128				4.80
Phylloquinone	Trp84 Ser82<br>Ser155<br>Ile153<br>Asp86<br>Asp97<br>Leu72<br>Leu76<br>Leu100<br>Ala59	Asn77	Leu57<br>Met89<br>Met135<br>Val75<br>Tyr80<br>Tyr88<br>Phe115<br>Phe126<br>Trp111<br>Ile99<br>Ala130	Leu85	–	5.26
Linoleic acid	Leu72<br>Ile135<br>Ser155	–	Val75<br>Met89<br>Met135	–	–	5.81

	Trp84<br>Asp97		Leu57<br>Leu85<br>Leu100<br>Tyr80<br>Tyr88<br>Ile99<br>Trp111<br>Ala130<br>Phe115<br>Phe126			
Myristic acid	Ile99<br>Ile153<br>Ser155<br>Phe115<br>Phe126<br>Ala130<br>Gly136<br>Asp97	Trp84<br>Met135	Tyr80<br>Tyr88<br>Leu57<br>Leu72<br>Leu85<br>Leu100<br>Ala59<br>Val75	Trp111	–	5.66
Lauric acid	Ile153<br>Phe115<br>Phe126<br>Met135<br>Ala130<br>Trp111<br>Ser155<br>Asp97<br>Tyr80	Trp84	Tyr88<br>Ile99<br>Leu57<br>Leu72<br>Leu85<br>Leu100<br>Val75<br>Ala59	–	–	5.57
Pentadecyclic acid	Asp97<br>Ser155<br>Trp111<br>Phe115<br>Phe126<br>Met135<br>Ala130	Trp84	Leu57<br>Leu72<br>Leu85<br>Leu100<br>Val75<br>Ile153<br>Ala59<br>Tyr80<br>Ile99	Tyr88	–	5.65
Palmitic acid	Asp97<br>Phe115	Trp84	Ile99<br>Leu57	–	–	5.34

	Phe126<br>Met135<br>Ala130<br>Ser155<br>Ile153		Leu72<br>Leu85<br>Leu100<br>Trp111<br>Tyr80<br>Tyr88<br>Ala59<br>Val75<br>Met89			
Oleic acid	Leu72<br>Ile153<br>Ser155<br>Trp84<br>Asp97	–	Val75<br>Met89<br>Met135<br>Leu57<br>Leu85<br>Leu100<br>Tyr80<br>Tyr88<br>Ile99<br>Trp111<br>Ala130<br>Phe115<br>Phe126	–	–	5.81
Behenic acid	Asp86<br>Ser82<br>Asn77<br>Leu72<br>Ile153<br>Ser155<br>Asp97<br>Thr140<br>Tyr80<br>Ala130<br>Phe115	–	Met89<br>Leu57<br>Leu85<br>Leu100<br>Val75<br>Trp111<br>Tyr88<br>Ile99	–	Trp84	5.31

	Phe126<br>Met135					
Tricosylic acid	Leu72<br>Leu100<br>Asp97<br>Phe115<br>Ser82<br>Ser155<br>Ile153<br>Trp84	Asn77	Trp111<br>Leu57<br>Leu85<br>Val75<br>Met89<br>Met135<br>Tyr80<br>Tyr88<br>Ile99<br>Phe126<br>Ala130	–	–	5.31
Arachidic acid	Trp84<br>Tyr80<br>Asp97<br>Ser155<br>Ile153<br>Ala59	Asn77	Met89<br>Met135<br>Ala130<br>Phe115<br>Phe126<br>Leu57<br>Leu72<br>Leu85<br>Leu100<br>Ile99<br>Tyr88<br>Val75	Trp111	–	5.60
Stearic acid	Phe115<br>Ile153<br>Asp86<br>Met135<br>Asp97	–	Trp111<br>Ala130<br>Leu57<br>Leu72<br>Leu85	–	–	5.64

	Trp84<br>Ser155		Leu100<br>Phe126<br>Ile99<br>Tyr80<br>Tyr88<br>Met89<br>Val75			
Lignoceric acid	Ser82<br>Ser155<br>Asn77<br>Asp97<br>Phe115<br>Phe126<br>Ala130<br>Met135<br>Trp111<br>Tyr80<br>Ile153	Trp84	Met89<br>Leu85<br>Val75<br>Ile99<br>Tyr88<br>Leu59<br>Leu72<br>Leu100	–	–	5.14
Capric acid	Ala59<br>Ala130<br>Phe115<br>Phe126<br>Ser155<br>Ile153<br>Leu72<br>Leu85<br>Leu100<br>Thr140<br>Asp97<br>Trp111	Trp84<br>Met135	Ile99<br>Tyr80<br>Tyr88<br>Leu57<br>Val75	–	–	5.83

Caprylic acid	Leu85<br>Phe115<br>Phe126<br>Ala130<br>Ser155<br>Asp97<br>Ile153	Trp84<br>Met135	Leu57<br>Leu100<br>Ile99<br>Tyr80<br>Trp111	Tyr88	–	5.44
Caproic acid	Trp84<br>Phe115<br>Leu57<br>Leu100<br>Tyr80<br>Thr140<br>Ile153	Tyr88<br>Asp97<br>Ser155	Ile99<br>Trp111<br>Ala130<br>Phe126<br>Met135	–	–	5.45

Table S8. Energy components (kcal/mol) after 100 ns MDS

Target	Ligands	ΔE_{vdW}	ΔE_{elec}	ΔG_{gas}	ΔG_{solv}	ΔG_{bind}
C. violaceum CviR +	Cinnamaldehyde	-20.82 ± 1.89	-9.93 ± 4.89	-30.75 ± 5.28	13.85 ± 3.48	-16.90 ± 2.43
	Azithromycin	-17.33 ± 4.99	-122.04±84.80	-153.02± 83.00	120.97±79.60	-32.05 ± 7.27
	Phylloquinone	-59.46 ± 3.72	-3.33 ± 2.89	-62.80 ± 4.56	12.23 ± 2.82	50.56 ± 3.70
	Linoleic acid	-42.80 ± 2.92	-18.19± 12.84	-61.00 ± 12.81	19.82 ± 9.80	-41.17 ± 4.05
	Myristic acid	-35.38 ± 2.82	-12.51± 2.45	-47.89 ± 3.45	14.70 ± 2.44	-33.19 ± 3.36
	Lauric acid	-31.66 ± 2.81	-12.67 ± 5.73	-44.34 ± 5.65	13.10 ± 2.51	-31.23 ± 3.99

ΔE_{vdW} = van der Waals energy; ΔG_{bind} = total binding free energy; ΔE_{gas} = gas phase free energy; ΔE_{elec} = electrostatic energy; ΔG_{sol} = solvation free energy.

Table S9. RMSD, ROG, RMSF, SASA, H–Bonds after 100 ns MDS

Target	Ligands	RMSD (Å)	RMSF(Å)	ROG (Å)	SASA (Å)	Intramolecular H–bond
C. violaceum CviR +	Apo CviR	2.23 ± 0.31	1.23 ± 0.59	16.02 ± 0.09	9873.76 ± 240.77	87.38 ± 6.39
	Cinnamaldehyde	1.76 ± 0.23	1.21 ± 0.58	16.16 ± 0.10	9985.89 ± 239.47	89.19 ± 6.86
	Azithromycin	1.80 ± 0.27	1.28 ± 1.20	16.37 ± 0.12	10590.29±298.77	91.00 ± 6.66
	Phylloquinone	1.55 ± 0.26	1.22 ± 0.66	16.24 ± 0.10	10035.65±254.61	90.14 ± 6.49
	Linoleic acid	2.63 ± 0.95	1.48 ± 1.58	16.29 ± 0.20	10092.75±298.38	90.00 ± 6.70
	Myristic acid	1.23 ± 0.12	1.04 ± 0.43	16.14 ± 0.09	9706.87±252.52	94.35 ± 6.54
	Lauric acid	1.34 ± 0.22	1.08 ± 0.57	16.17 ± 0.10	9973.64±263.54	91.43 ± 6.61

RMSD = Root mean square deviation; RMSF = root mean square fluctuations; RoG = radius of gyration (RoG); SASA = solvent accessible surface area.

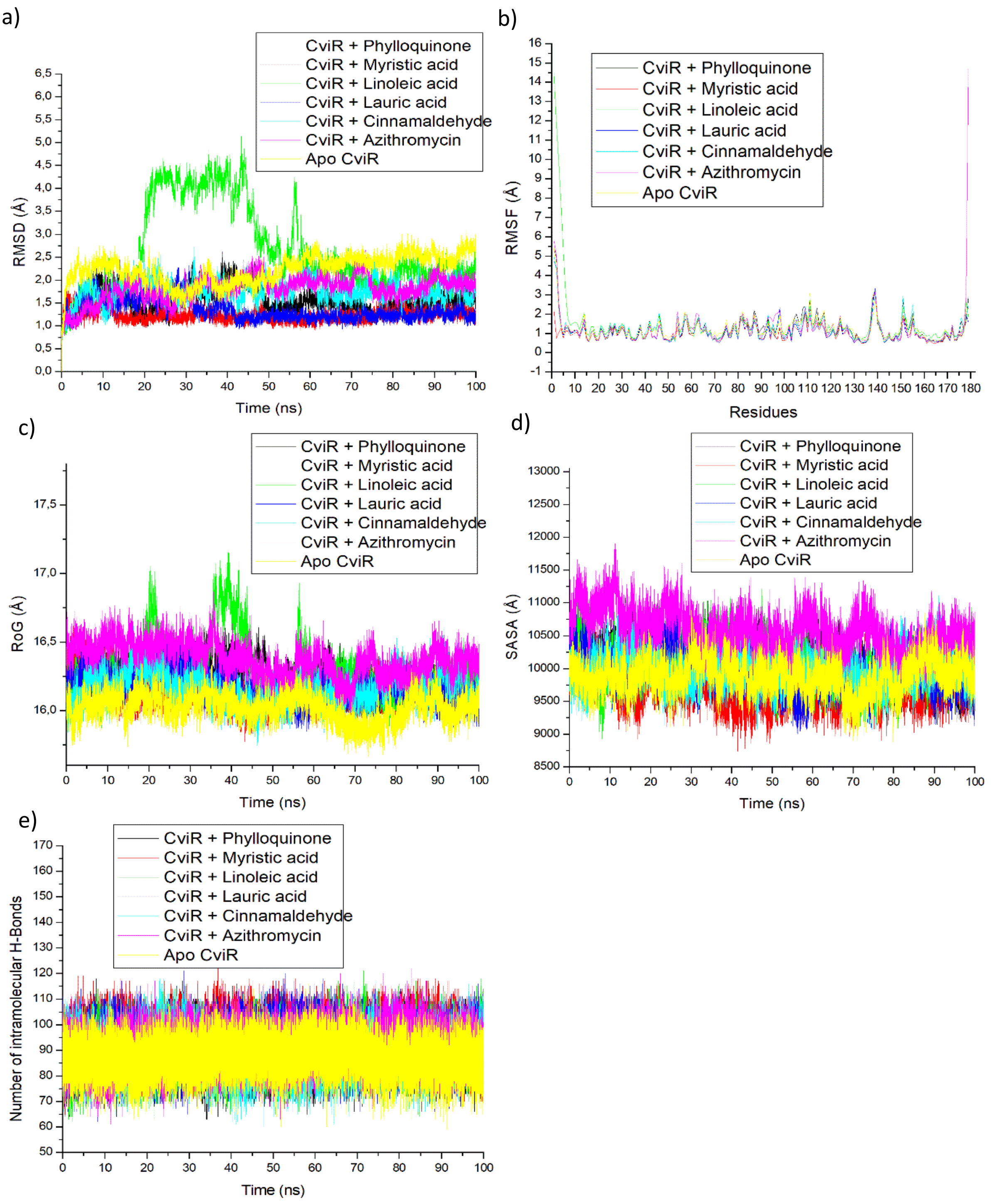

Figure S4. (a) Root mean square deviation (RMSD), (b) root mean square fluctuations (RMSF), (c) radius of gyration (RoG), (d) solvent accessible surface area (SASA) and (e) number of intramolecular hydrogen bonds with time plots of comparison between CviR and top four compounds, azithromycin and cinnamaldehyde determined over 100 ns molecular dynamics simulations.

Table S10: Plots of interactions of top-ranked metabolites and reference standard towards LasR and CviR at different time interval during the 100 ns MD simulation.

Complex	Duration	2D Interactions
LasR+ Cinnamaldehyde	30 ns	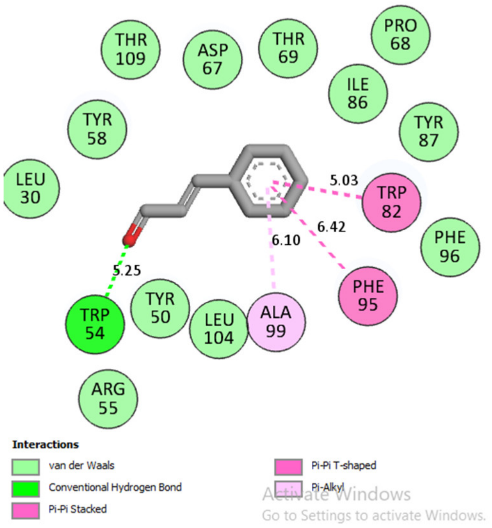
	60 ns	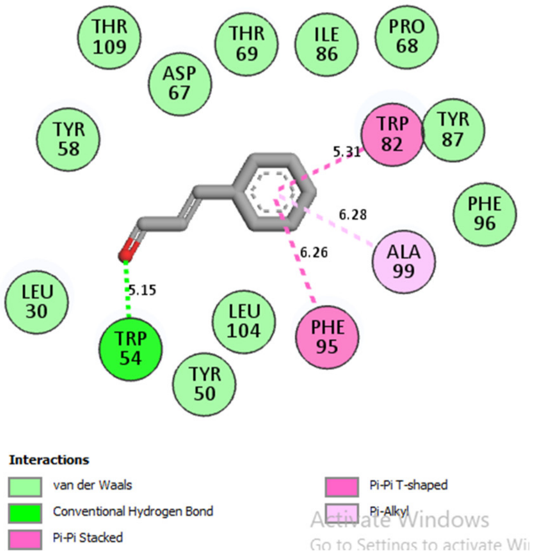

	100 ns	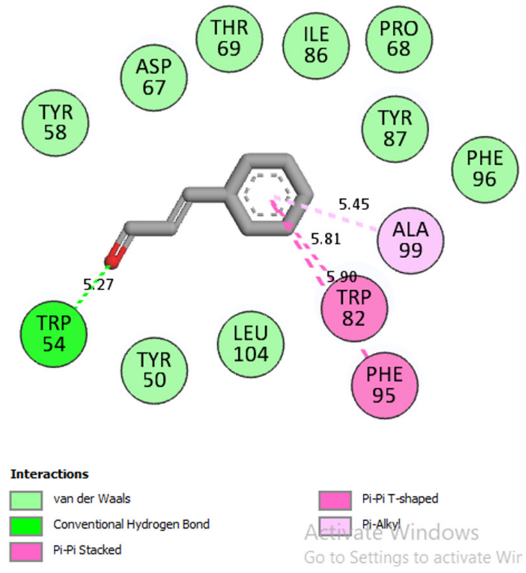
LasR+ Azithromycin	30 ns	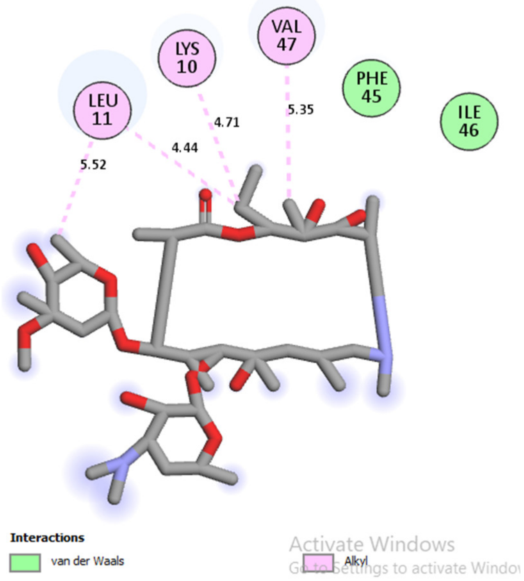
	60 ns	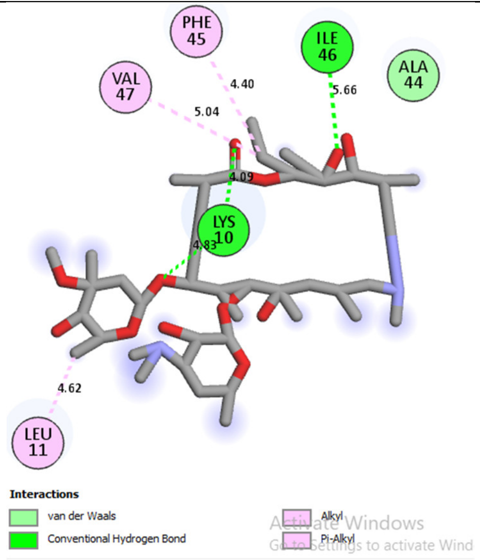

	100 ns	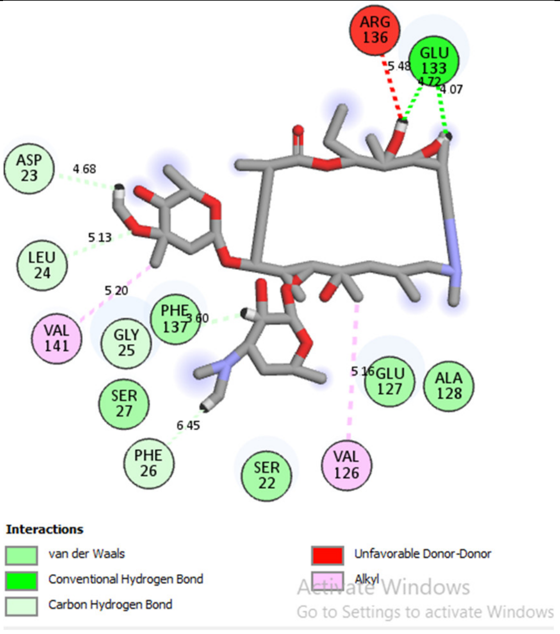
LasR+ Phylloquinone	30 ns	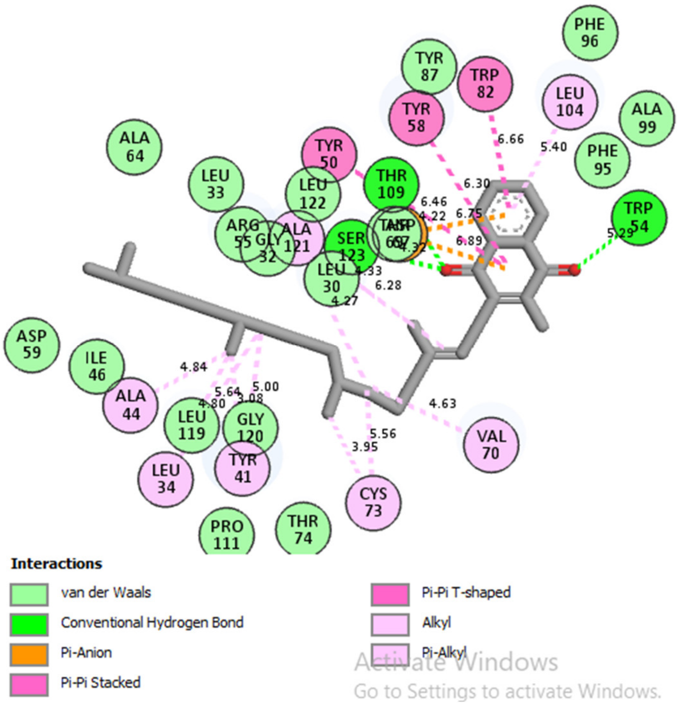

	60 ns	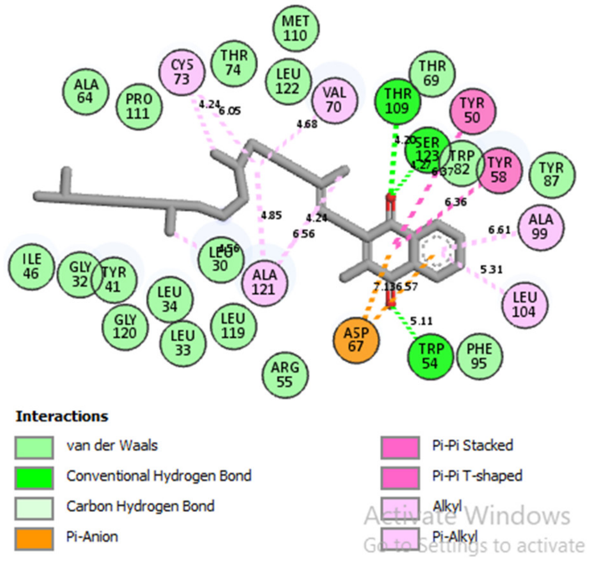 MET 110<br>CYS 73<br>THR 74<br>LEU 122<br>VAL 70<br>THR 69<br>ALA 64<br>PRO 111<br>THR 109<br>TYR 50<br>SER 123<br>TRP 82<br>TYR 58<br>TYR 87<br>ALA 99<br>ILE 46<br>GLY 32<br>TYR 41<br>LEU 30<br>ALA 121<br>LEU 34<br>GLY 120<br>LEU 33<br>LEU 119<br>LEU 104<br>ASP 67<br>TRP 54<br>PHE 95<br>ARG 55<br>Interactions<br>van der Waals<br>Conventional Hydrogen Bond<br>Carbon Hydrogen Bond<br>Pi-Anion<br>Pi-Pi Stacked<br>Pi-Pi T-shaped<br>Alkyl<br>Pi-Alkyl
	100 ns	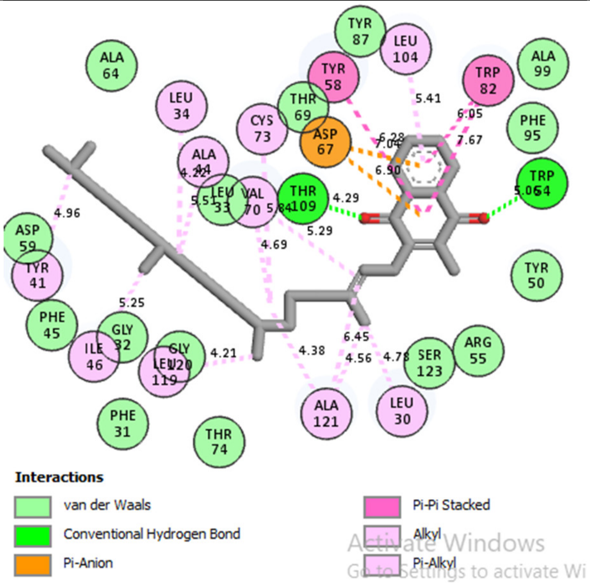 TYR 87<br>LEU 104<br>ALA 64<br>TYR 58<br>TRP 82<br>ALA 99<br>LEU 34<br>THR 69<br>CYS 73<br>ASP 67<br>PHE 95<br>ALA 44<br>LEU 33<br>VAL 70<br>THR 109<br>TRP 64<br>ASP 59<br>TYR 41<br>TYR 50<br>PHE 45<br>GLY 32<br>ILE 46<br>GLY 120<br>LEU 119<br>SER 123<br>ARG 55<br>ALA 121<br>LEU 30<br>PHE 31<br>THR 74<br>Interactions<br>van der Waals<br>Conventional Hydrogen Bond<br>Pi-Anion<br>Pi-Pi Stacked<br>Alkyl<br>Pi-Alkyl

<table>
<tr><td rowspan="2">LasR+ Linoleic acid</td><td>30 ns</td><td>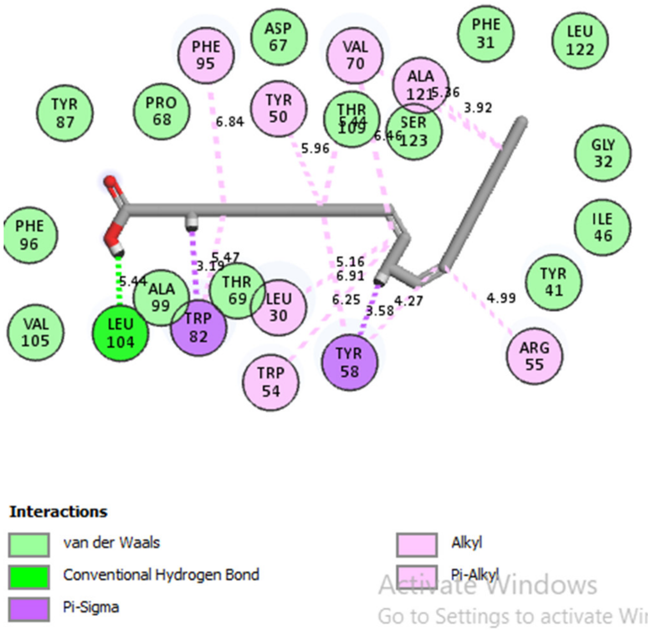
</td></tr>
<tr><td>60 ns</td><td>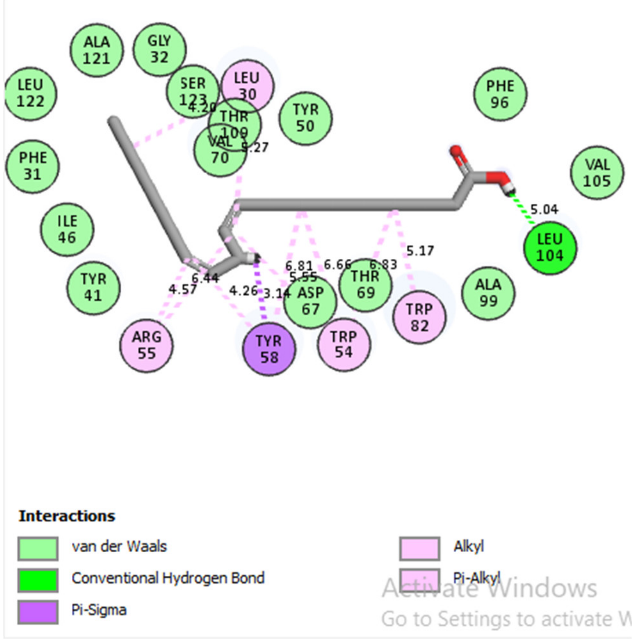
</td></tr>
</table>

	100 ns	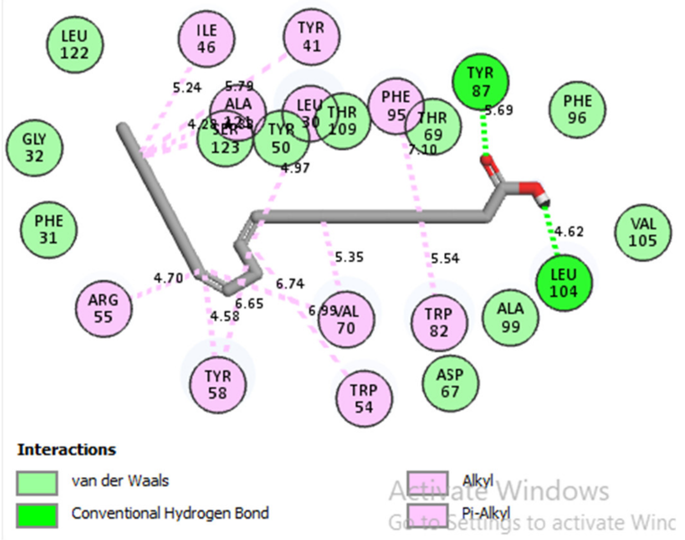
LasR+ Oleic acid	30 ns	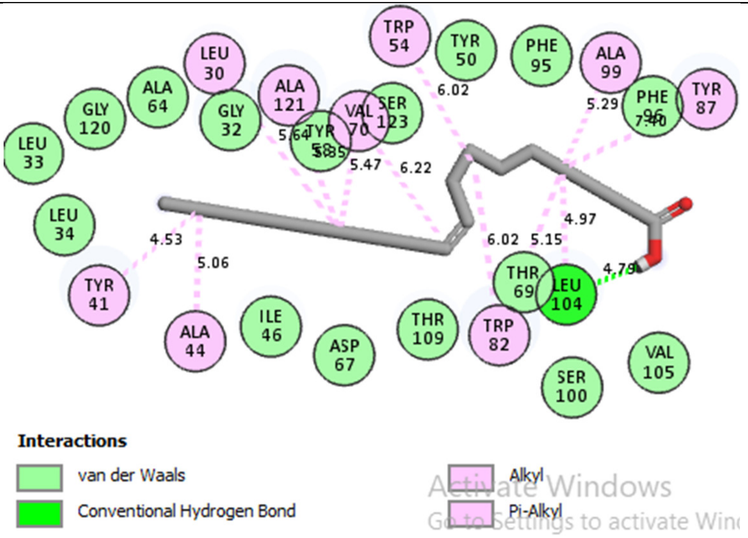
	60 ns	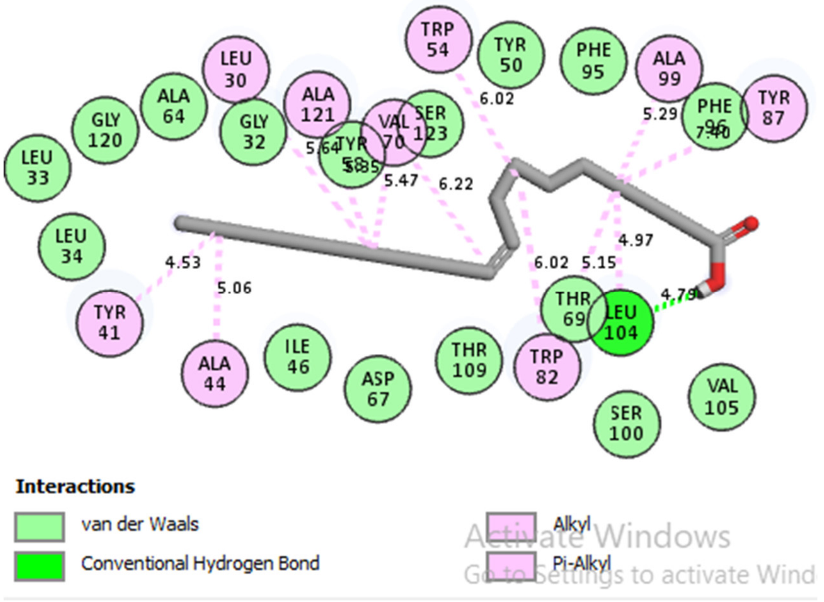

<table>
<tr><td></td><td>100 ns</td><td>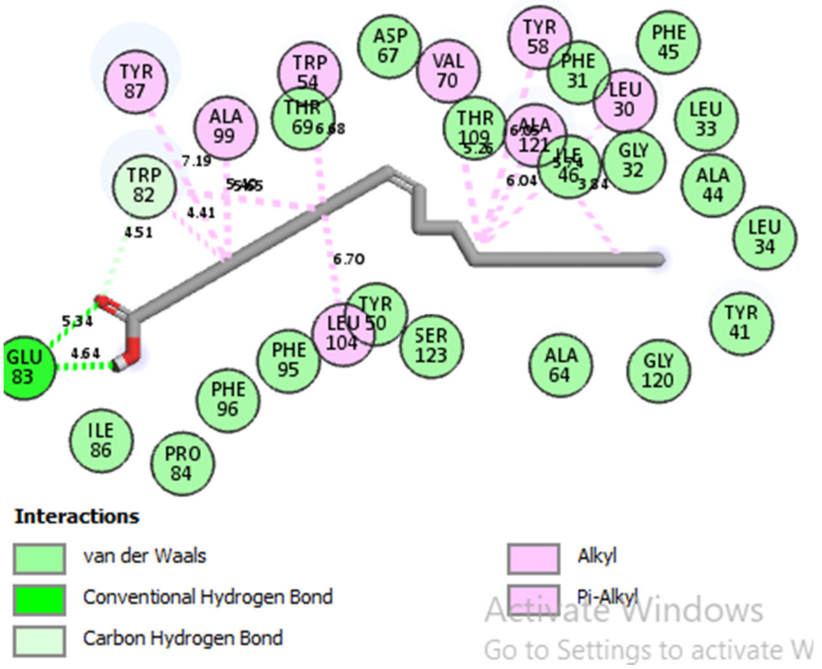
</td></tr>
<tr><td>CviR + Cinnamaldehyde</td><td>30 ns</td><td>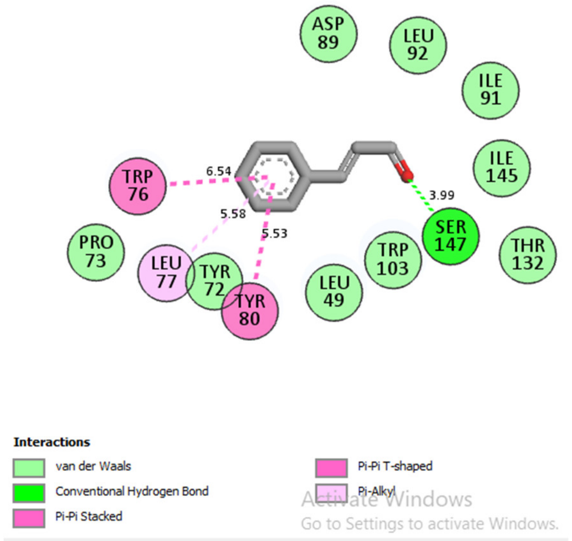
</td></tr>
</table>

<table>
<tr><td></td><td>60 ns</td><td>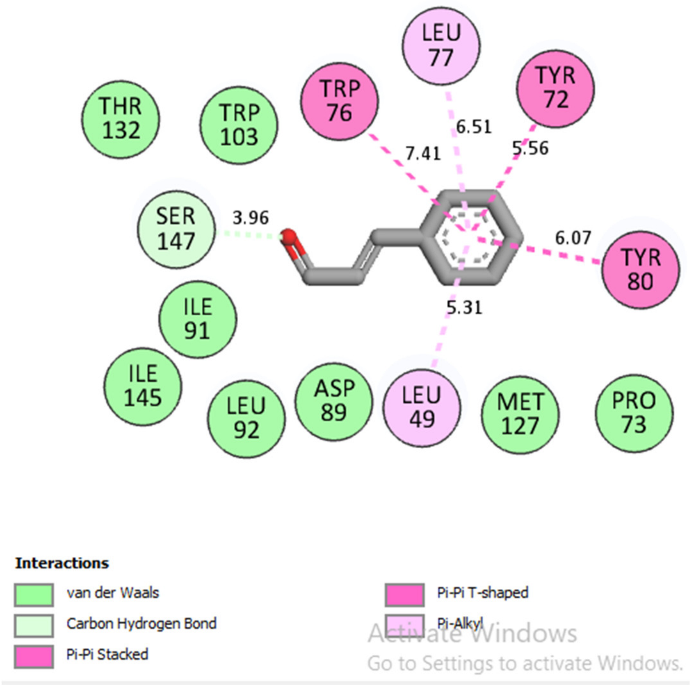
</td></tr>
<tr><td></td><td>100 ns</td><td>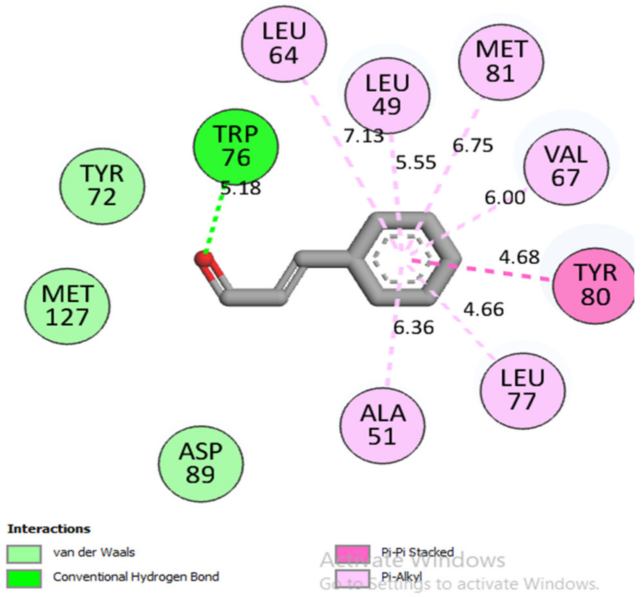
</td></tr>
</table>

<table>
<tr><td rowspan="2">CviR + Azithromycin</td><td>30 ns</td><td>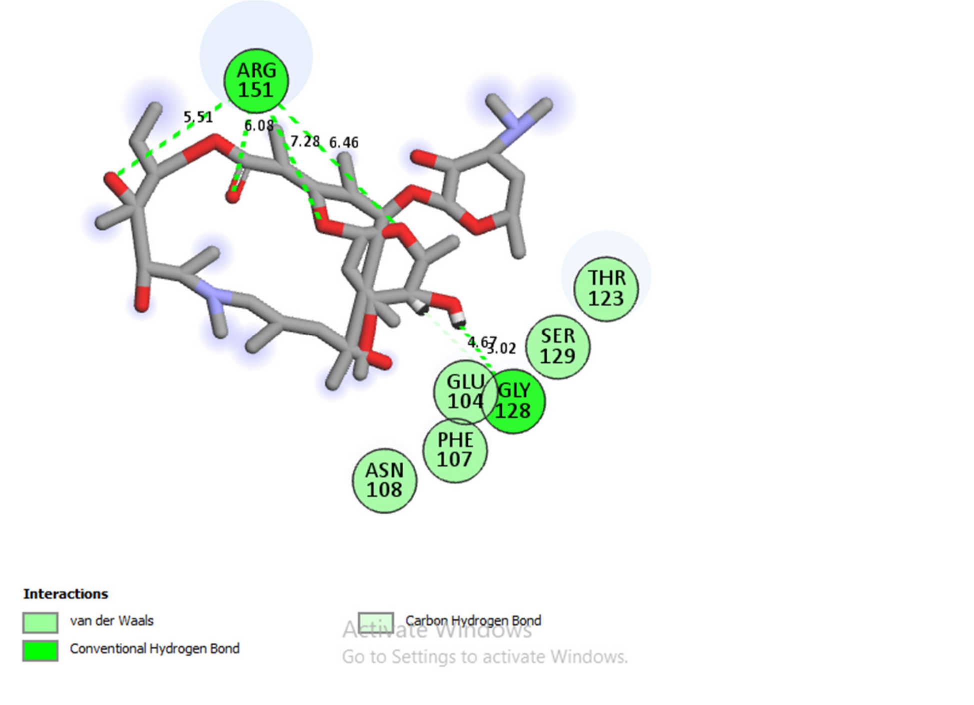
</td></tr>
<tr><td>60 ns</td><td>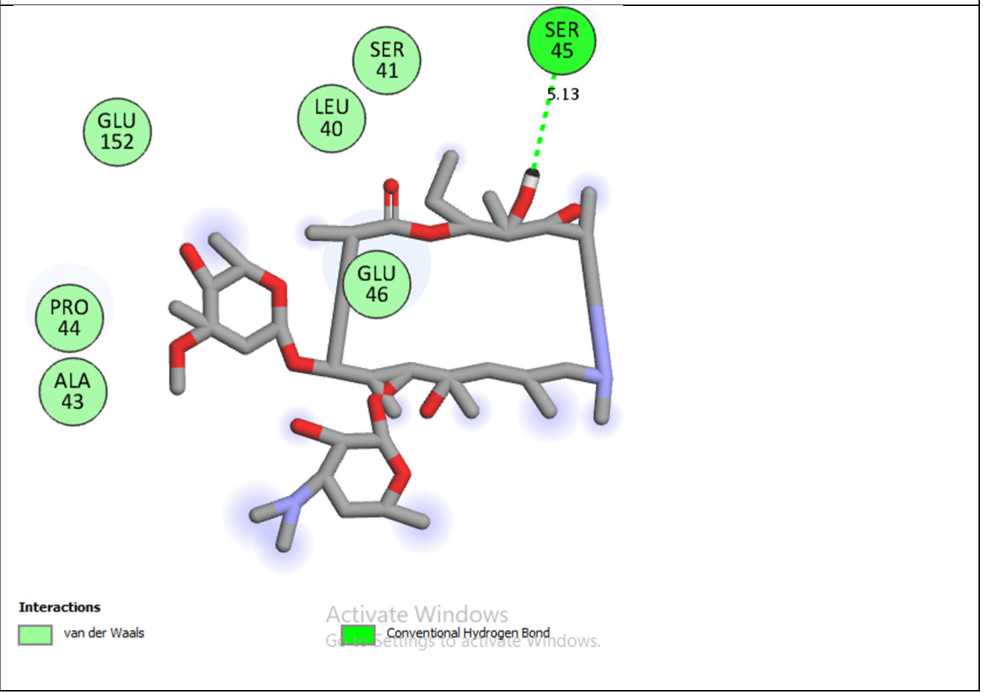
</td></tr>
</table>

<table>
<tr><td></td><td>100 ns</td><td>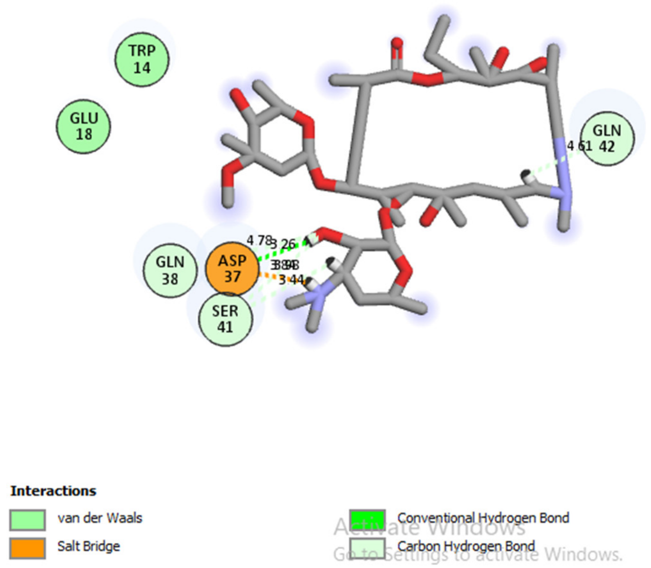
</td></tr>
<tr><td>CviR+
Phylloquinone</td><td>30 ns</td><td>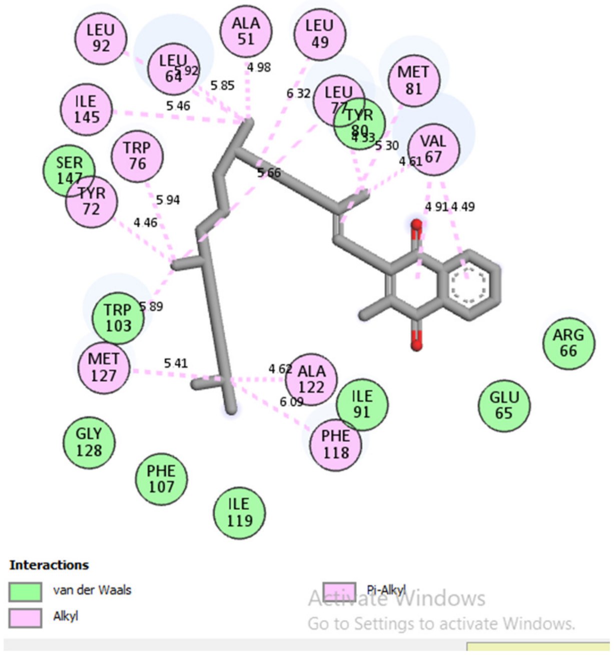
</td></tr>
</table>

<table>
<tr><td></td><td>60 ns</td><td>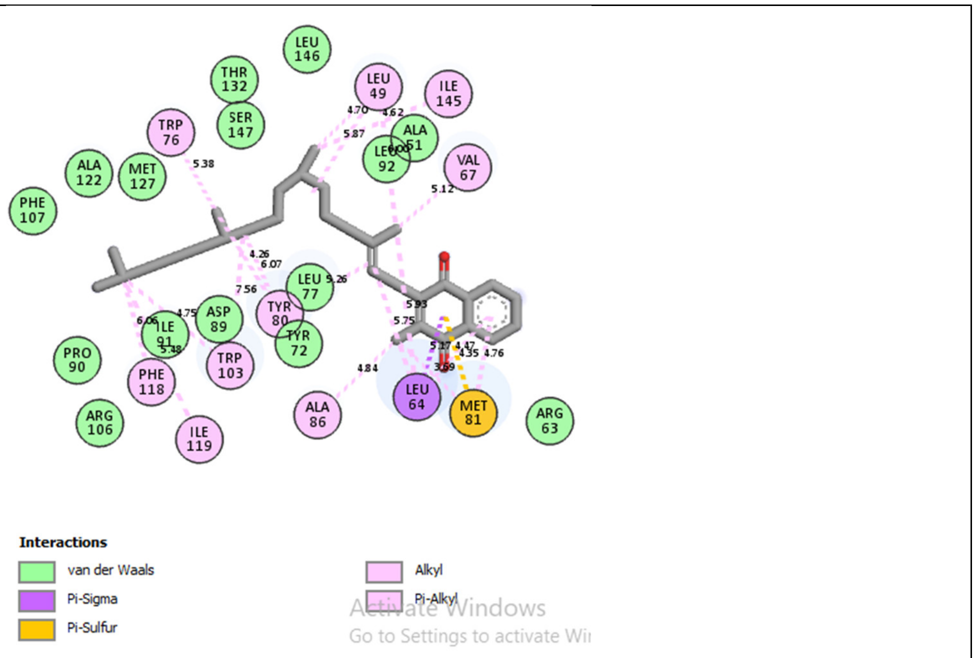
</td></tr>
<tr><td></td><td>100 ns</td><td>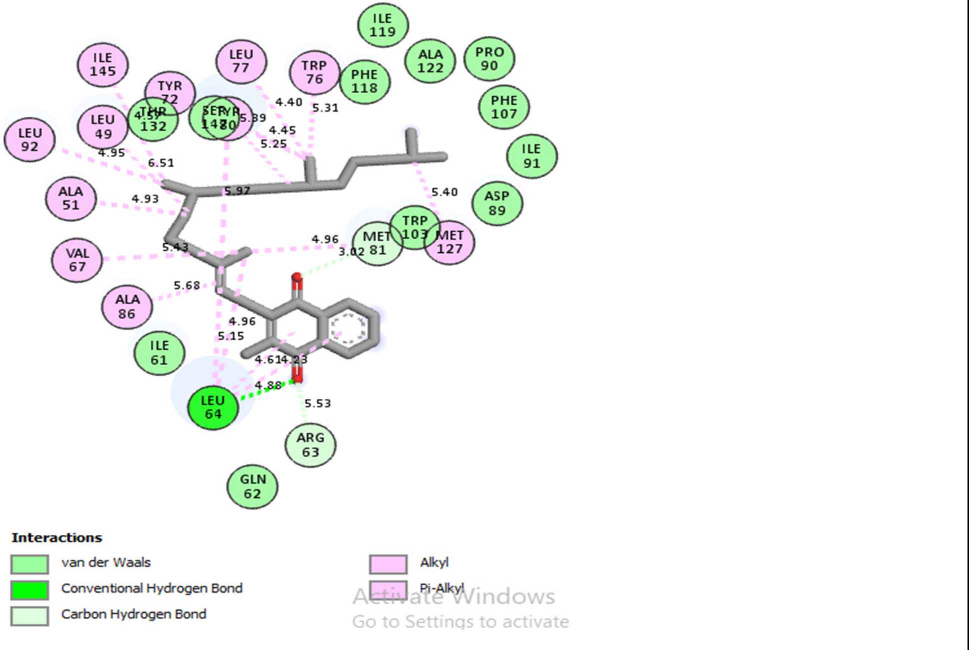
</td></tr>
</table>

<table>
<tr><td rowspan="2">CviR + Linoleic acid</td><td>30 ns</td><td>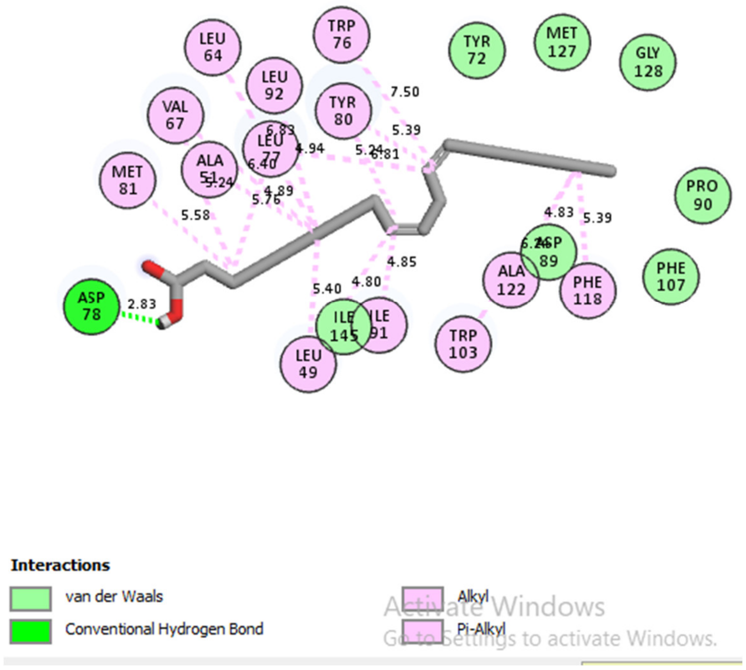
</td></tr>
<tr><td>60 ns</td><td>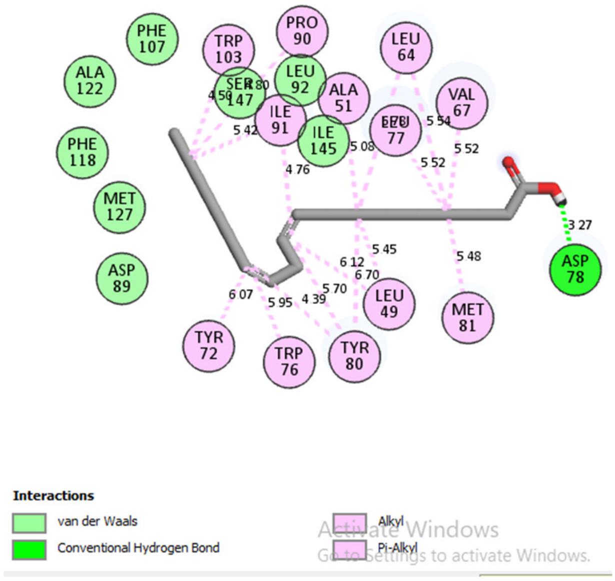
</td></tr>
</table>

<table>
<tr><td></td><td>100 ns</td><td>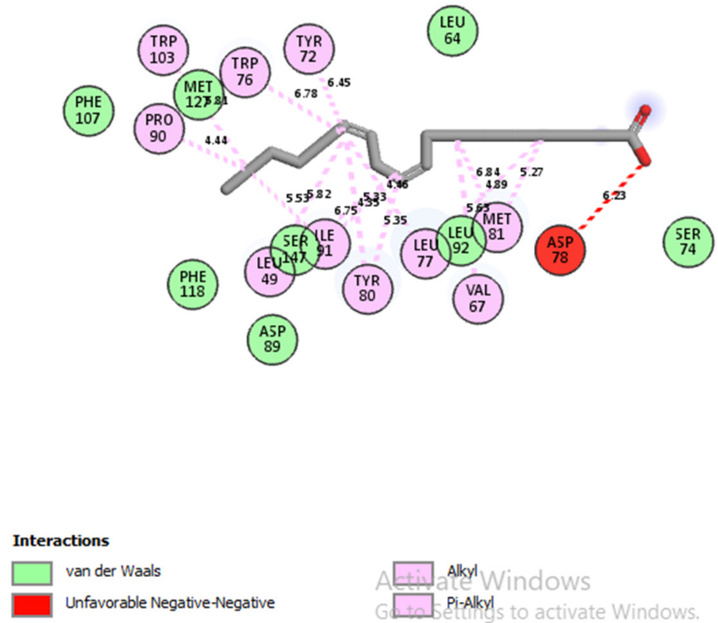
</td></tr>
<tr><td>CviR + Myristic acid</td><td>30 ns</td><td>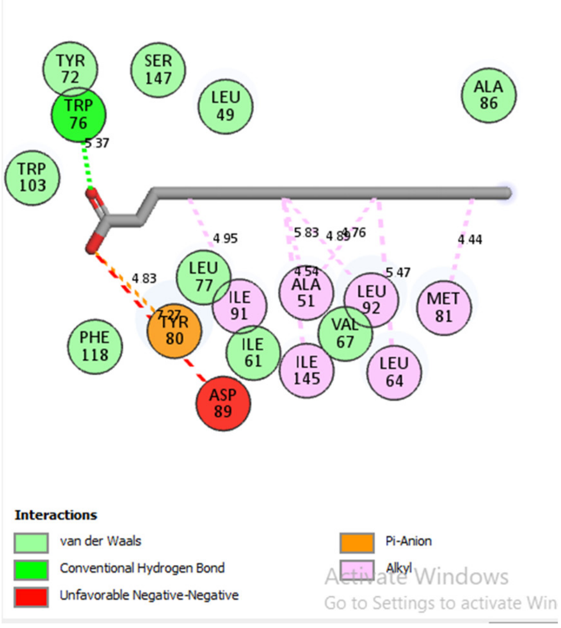
</td></tr>
</table>

<table>
<tr><td></td><td>60 ns</td><td>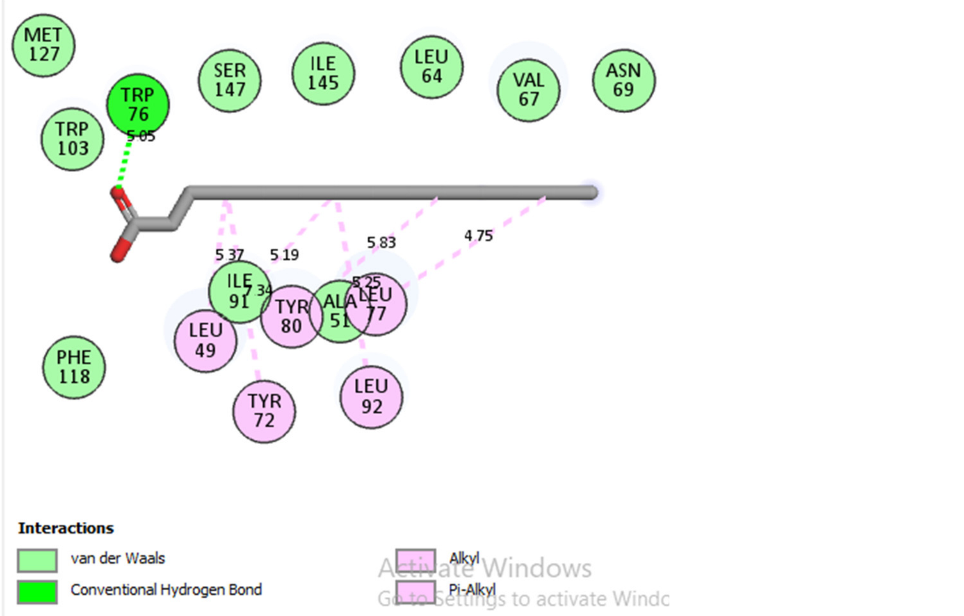
</td></tr>
<tr><td></td><td>100 ns</td><td>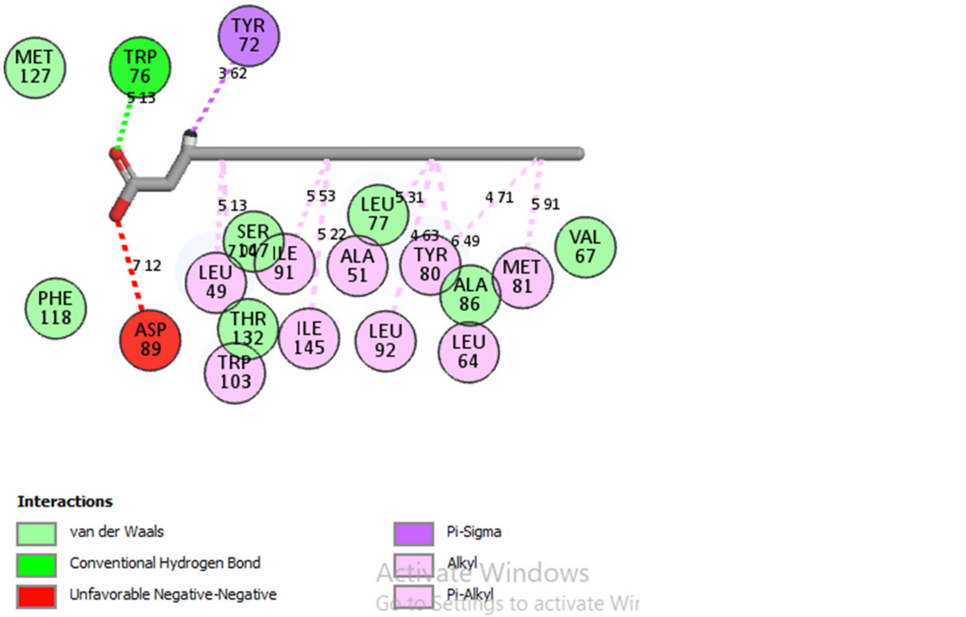
</td></tr>
</table>

<table>
<tr><td rowspan="2">CviR + Lauric acid</td><td>30 ns</td><td>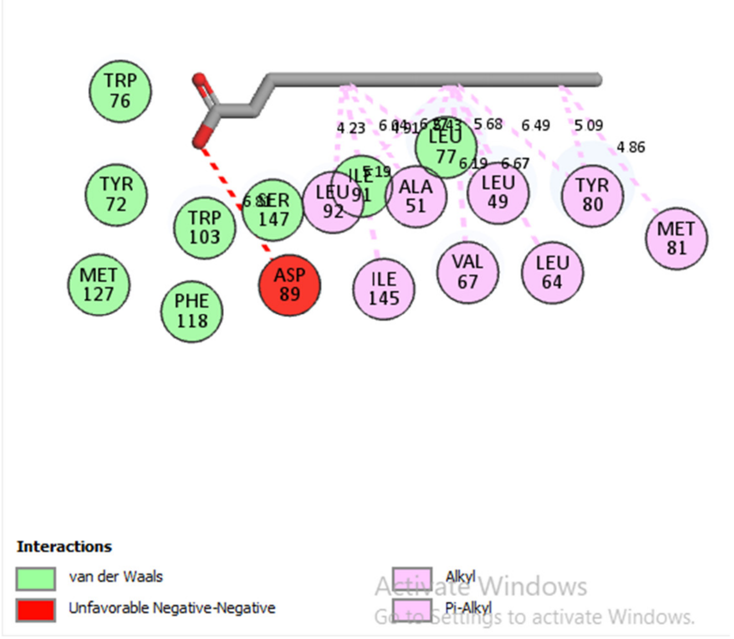
</td></tr>
<tr><td>60 ns</td><td>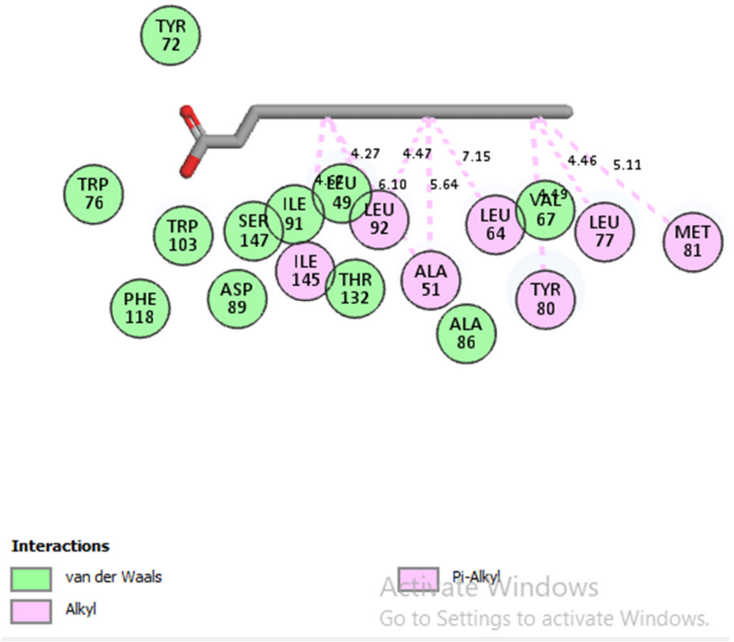
</td></tr>
</table>

<table>
<tr><td></td><td>100 ns</td><td>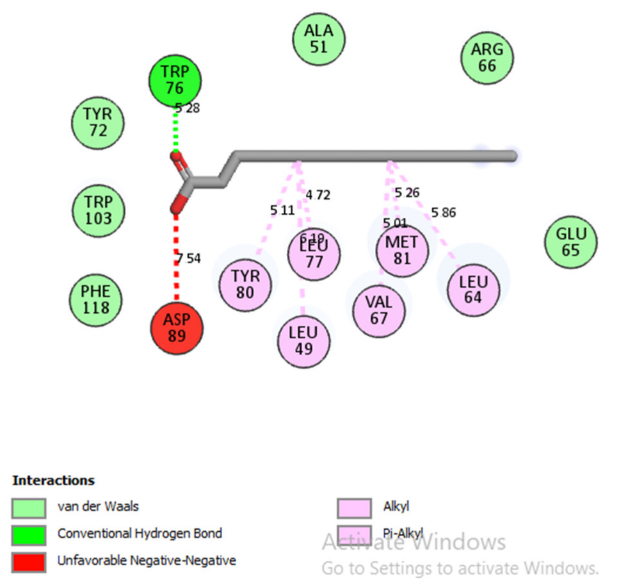
</td></tr>
</table>
